# *Escherichia* cryptic clade I is an emerging source of human intestinal pathogens

**DOI:** 10.1186/s12915-023-01584-4

**Published:** 2023-04-13

**Authors:** Miki Okuno, Yoko Arimizu, Seina Miyahara, Yuki Wakabayashi, Yasuhiro Gotoh, Shuji Yoshino, Tetsuya Harada, Kazuko Seto, Takeshi Yamamoto, Keiji Nakamura, Tetsuya Hayashi, Yoshitoshi Ogura

**Affiliations:** 1 Division of Microbiology, Department of Infectious Medicine, Kurume University School of Medicine, Kurume, Fukuoka 830-0011 Japan; 2 Department of Bacteriology, Graduate School of Medical Sciences, Kyushu University, Fukuoka, 812-8582 Japan; 3 Department of Infectious Disease, National Hospital Organization Kyushu Medical Center, Fukuoka, 810-0065 Japan; 4 Department of Microbiology, Miyazaki Prefectural Institute for Public Health and Environment, Miyazaki, 889-2155 Japan; 5 Division of Microbiology, Osaka Institute of Public Health, Osaka, 537-0025 Japan; 6 Division of Planning, Osaka Institute of Public Health, Osaka, 537-0025 Japan

**Keywords:** *Escherichia coli*, *Escherichia* cryptic clade I, Genome, Evolution, Shiga toxin, Heat-labile enterotoxin, Heat-stable enterotoxin

## Abstract

**Background:**

Within the genus *Escherichia*, several monophyletic clades other than the traditionally defined species have been identified. Of these, cryptic clade I (C-I) appears to represent a subspecies of *E. coli*, but due to the difficulty in distinguishing it from *E. coli *sensu stricto, the population structure and virulence potential of C-I are unclear.

**Results:**

We defined a set of true C-I strains (*n* = 465), including a Shiga toxin 2a (Stx2a)-producing isolate from a patient with bloody diarrhoea identified by the retrospective analyses using a C-I-specific detection system. Through genomic analysis of 804 isolates from the cryptic clades, including these C-I strains, we revealed their global population structures and the marked accumulation of virulence genes and antimicrobial resistance genes in C-I. In particular, half of the C-I strains contained hallmark virulence genes of Stx-producing *E. coli* (STEC) and/or enterotoxigenic *E. coli* (ETEC). We also found the host-specific distributions of virulence genes, which suggests bovines as the potential source of human infections caused by STEC- and STEC/ETEC hybrid-type C-I strains, as is known in STEC.

**Conclusions:**

Our findings demonstrate the emergence of human intestinal pathogens in C-I lineage. To better understand the features of C-I strains and their infections, extensive surveillance and larger population studies of C-I strains are needed. The C-I-specific detection system developed in this study will be a powerful tool for screening and identifying C-I strains.

**Supplementary Information:**

The online version contains supplementary material available at 10.1186/s12915-023-01584-4.

## Background

*Escherichia* cryptic clade I (C-I) is one of the six cryptic clades proposed in the genus *Escherichia* [1–3]. Among these, clades III and IV have recently been defined as a separate species, *E. ruysiae* [4] [referred to as Er (C-III) and Er (C-IV) in this manuscript, respectively], and clade V was classified as *E. marmotae* [5] [Em (C-V)]. A new species, “*E. whittamii*”, was also recently proposed for clade II [6] [C-II (“Ew”)]. Although the taxonomic position of C-I has not yet been fully defined, several phylogenetic analyses have shown that C-I is closely related to *E. coli *sensu stricto (*E. coli ss*) and could represent a subspecies of *E. coli* [2, 3, 7–9]. Based on whole-genome sequence analysis, C-II (“Ew”), Er (C-III/IV), and Em (C-V) have been identified within discrete clusters of 95% average nucleotide identity (ANI) [10, 11], while C-I is grouped with *E. coli* into a 95% ANI cluster [12].

Among three common *Escherichia* species, namely, *E. coli, E. albertii* and *E. fergusonii* (note that reassignment of *E. hermannii* [13] to “*Atlantibacter hermannii*” was recently proposed [14]), *E. coli* is a genetically and phenotypically diverse group of strains, ranging from nonpathogenic commensal strains to pathogenic strains of various pathotypes that cause intestinal and extraintestinal diseases in humans and animals. Each pathotype possesses specific virulence factors introduced by mobile genetic elements (MGEs) [15]. Although *E. albertii* and *E. fergusonii* are not frequently isolated from humans [16],* E. albertii* has been recently recognized as a human enteric pathogen possessing a type III secretion system (T3SS) similar to that of enteropathogenic *E. coli* (EPEC) and major Shiga toxin (Stx)-producing *E. coli* (STEC) [17–19]. *E. fergusonii* causes sporadic cases of bacteraemia, urinary tract infections and diarrhoea, but its virulence determinants remain unknown [16].

While many of the strains belonging to cryptic clades or recently defined *Escherichia* species are often identified as *E. coli* in clinical laboratories due to the difficulty in accurately identifying these strains by routine diagnostic biochemical examinations C-II (“Ew”), Er (C-III/IV) and Em (C-V) strains are isolated mainly from environmental samples, such as water, wastewater and soil [2]. In addition, these strains carry no virulence genes or only minor potential virulence genes. In contrast, C-I strains are frequently isolated from the faeces of humans and other animals and sometimes from children with diarrhoea. Several strains producing Stx, heat-labile enterotoxin (LT) and/or heat-stable enterotoxin (ST), the hallmark virulence factors of STEC and enterotoxigenic *E. coli* (ETEC), respectively [15], have been isolated from animals [1, 2, 20, 21]. C-I strains producing ETEC enterotoxins have also been isolated from human patients [2, 22]. However, the relevance of such C-I strains in the C-I population and the virulence potential of these strains are unknown. The global population structure of C-I has also yet to be elucidated. This lack of knowledge is at least partly due to the difficulty in accurately identifying C-I strains [2]. Although a rapid PCR-based method have been recently developed to identify cryptic clades, the two-step screening strategy employed in this typing method is unsuitable for extensive screening purposes, and a possibility of false identification of C-I has also been indicated [7].

In this study, we performed an extensive database search to accurately define C-I genomes and developed a one-step PCR-based C-I detection system. This system was successfully applied in retrospective analyses to identify C-I strains among strains previously identified as *E. coli*, including an *stx*-positive C-I strain isolated from a patient suffering from bloody diarrhoea. We further performed a phylogenomic analysis of accurately defined C-I strains, along with recently defined *Escherichia* species and other cryptic clades, and showed, in addition to the global population structure of C-I and its phylogenetic relationship to *E. coli ss*, the marked accumulation of *E. coli* virulence genes and antimicrobial resistance (AMR) genes in C-I. In particular, half of the C-I strains in the dataset contained hallmark virulence genes of STEC and/or ETEC. These strains were classified into STEC, ETEC and STEC/ETEC hybrid types, and bovines are suggested as a potential source of human infection of STEC- and STEC/ETEC hybrid-type strains. Data showing genetic links between the accumulation of *E. coli* virulence genes and MGEs in the C-I lineage and the accumulation of more AMR genes in nonintestinal pathogenic strains are also presented.

## Results

### Identification of C-I genomes in public databases and development of C-I-specific PCR

Since cryptic clades and recently defined *Escherichia* species are usually identified as *E. coli* in routine bacterial identification [2], most of their genome sequences were assumed to be registered as *E. coli* sequences in public databases. The EnteroBase database includes all complete genome sequences and Illumina paired-end reads deposited in the NCBI database and original sequence data uploaded by users for several bacterial genera/species, including *Escherichia* spp., and raw read sequences are de novo assembled using a standardized pipeline [23]. Genotyping data for *Escherichia* spp. based on several tools, including ClermonTyping [24] and EzClermont [25], are also available in EnteroBase. To identify strains belonging to cryptic clades and recently defined *Escherichia* species in the Enterobase database, we obtained a total of 1088 genomes that were not deposited as *E. albertii* or *E. fergusonii* and were not assigned to any of the *E. coli* phylogroups as determined by both ClermonTyping and EzClermont. After filtering out low-quality data, 1065 genomes were used for constructing a core gene-based phylogenomic tree (Additional file 1: Fig. S1). In this tree, all strains categorized as “clade I” (*n* = 453), “clade II” (*n* = 5), “clade III” (*n* = 49), “clade IV” (*n* = 46) and “clade V” (*n* = 213) by ClermonTyping formed deep-branching lineages. Strains categorized as “clade III” and “clade IV” strains formed sister lineages as expected, and lineages categorized as “clade IV” and “clade V” included the type strains of Er and Em, respectively. Although strains deposited as C-VI (*n* = 2) or C-VIII (*n* = 5) were categorized as *E. coli* phylogroup F by ClermonTyping, these strains formed two distinct deep-branching lineages (Additional file 1: Fig. S1). Both strains deposited as C-VII (categorized as “clade II” and “unknown” in ClermonTyping) clustered with C-II (“Ew”) strains; thus, they were regarded as C-II (“Ew”) in this study. All the strains categorized as “E or clade I” (*n* = 133) and almost all the strains categorized as “unknown” (41 out of 43) by ClermonTyping clustered with strains categorized as *E. coli* phylogroups “A” and “G”, which apparently belong to the *E. coli ss* lineage. In EzClermont typing, many strains categorized as phylogroups “A” (157 out of 181) and “E” (202 out of 210) were grouped into the C-I lineage. Conversely, a considerable number of strains categorized as “cryptic” (127 out of 194) and “U/cryptic” (111 out of 437) were grouped into the *E. coli ss* or *E. fergusonii* lineage. EzClermont and ClermonTyping employ in silico PCR analysis using eight and fifteen primer pairs, respectively [24, 25]. The difference in primer sets was thought to be the primary cause of the variation in accuracy between these two systems for the classification of cryptic clades. Finally, we identified 453 C-I genomes in the *Escherichia* genomes deposited in EnteroBase. In addition, 6, 50, 46, 213, 2 and 5 genomes were identified as C-II (“Ew”), Er (C-III), Er (C-IV), Em (C-V), C-VI and C-VIII, respectively (Additional file 2: Table S1).

Using these genomes, we performed a pangenome-wide association study to search for C-I-specific genes that can be used for developing a PCR-based C-I detection system for efficient screening of C-I strains. In this analysis, we included 17 C-II genomes that were available in the NCBI database but not in EnteroBase [9] and representative strains of *E. albertii*, *E. fergusonii* and eight *E. coli* phylogroups (A to G) (Additional file 2: Table S1). This analysis identified one gene (1368 bp in length, of unknown function with two secreted effector protein pipB2 domains) that was conserved in nearly all the C-I strains (452 out of 453) and completely absent in the other strains analysed. The C-I-specific gene (locus_tag: EsCd1-10290c_1484 in strain 10,290) was located on putative genomic island, and no homologue gene (> 60% amino acid identity) was detected in other members of the *Enterobacteriaceae* family*.* We designed a primer pair for this gene and developed a PCR system to detect C-I strains. We confirmed that this gene is not present in any of the 1134 *E. coli* strains that we previously isolated from humans and animals and sequenced [26]. To evaluate the specificity of this PCR system, we first performed in silico PCR analyses of these 1134 *E. coli* strains and confirmed that all yielded negative results. We further performed PCR and in silico PCR analyses of seven C-I strains that were isolated from healthy bovine stools during our abovementioned study on *E. coli* strains [26] and defined as belonging to C-I based on their genome sequences. In this analysis, all the strains yielded a PCR product of the expected size (Additional file 1: Fig. S2). These analyses confirmed the specificity of the PCR system that we developed. Notably, the seven C-I strains were all *stx2*-positive; five carried *stx2a*, and two carried *stx2g* (Additional file 2: Table S1).

### Retrospective screening of C-I strains in the “*E. coli*” strains isolated from clinical, food and animal samples

Using the PCR-based C-I detection system, we performed two series of retrospective screenings of C-I strains. In the first screening, we analysed strains isolated from human patients with intestinal diseases and identified as *E. coli* exhibiting minor serotypes or O-untypeable in Miyazaki (*n* = 41) and Osaka (*n* = 207) prefectures, Japan, from 1995 to 2014. This screening identified two PCR-positive strains (EC05-109 and 10290). The former was isolated from a patient with diarrhoea in Osaka in 2005 and exhibited the O1:H45 serotype. This strain carried none of the hallmark virulence genes of the five major pathotypes of intestinal pathogenic *E. coli*, including *stx* for STEC, *elt* and *est* for ETEC, *eaeA* for EPEC, *aggR* for enteroaggregative *E. coli* (EAEC) and *ipaH* for enteroinvasive *E. coli* (EIEC). In addition, only one colony was detected in the specimen in routine diagnostic analysis; thus, this strain was unlikely to be the causative agent of the infection. However, strain 10290 showing the O2:H28 serotype, which was isolated from a 27-year-old man who was hospitalized with severe symptoms, including bloody diarrhoea, in Miyazaki in 2002, carried not only *stx2a* but also *esta* and *elt2* encoding STa and LT2, respectively*.* No other suspected intestinal pathogens were detected in the specimen from this patient. Furthermore, a strain isolated from the food (a variety of beef products) consumed by the patient exhibited an identical pulsed-field gel electrophoresis pattern as that of strain 10290. Thus, strain 10290 was considered the causative agent of the infection.

In the second screening, we analysed 18 strains exhibiting minor serotypes or O-untypeable strains in our laboratory *E. coli* collection isolated from foods, animals and the environment between 2007 and 2015 in Japan. This analysis also identified one food and two bovine C-I isolates. These three strains carried *stx2a* and *elt2a*, and one strain (strain PV14-161) additionally carried *esta4* among the hallmark virulence genes of intestinal pathogenic *E. coli*.

### Re-evaluation of the phylogenetic position of C-I in the genus *Escherichia* and the characteristic isolation sources and serotypes of these strains in the *E. coli* O:H serotyping scheme.

**Fig. 1:**
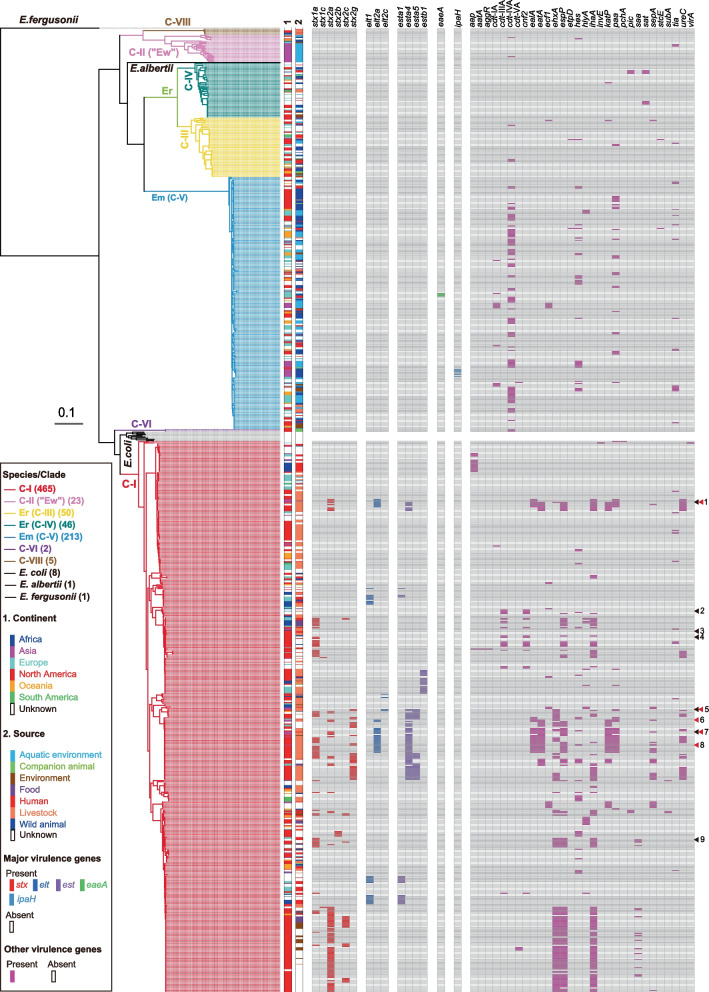
Phylogenomic relationships of C-I with *E. coli* ss, other *Escherichia* cryptic clades and recently defined *Escherichia* species and the distribution of virulence genes in these clades and species. The ML tree of 804 isolates from cryptic clades with 10 reference strains was constructed based on 339,491 SNP sites located on 1581 core genes. *E. fergusonii* ATCC_35469T, *E. albertii* EC06-170, *E. coli* MG1655 (phylogroup A), *E. coli* SE11 (phylogroup B1), *E. coli* SE15 (phylogroup B2), *E. coli* APECO78 (phylogroup C), *E. coli* UMN026 (phylogroup D), *E. coli* CB9615 (phylogroup E), *E. coli* SMS-3–5 (phylogroup F) and *E. coli* PSUO78 (phylogroup G) were included as references. Strains belonging to cryptic clades and recently defined species are highlighted with differently coloured lines. Information about the continents and sources of isolation and the distribution of virulence genes for each strain without references is also provided. Strains for which complete genome sequences and Stx2 production levels were determined are indicated by black and red arrowheads, respectively (1: KS-P079, 2: EC42405, 3: 3057, 4: 89–3506, 5: HH-P024, 6: SI-P041, 7: 10,290, 8: KS-P062 and 9: 2013C-4282)

Following the genome sequencing of the five C-I strains identified in the PCR screening, we constructed a core gene-based phylogenomic tree of the 465 C-I strains, including the 12 C-I strains that we sequenced and the 339 strains belonging to C-II (“Ew”), Er (C-III/IV), Em (C-V), C-VI or C-VIII obtained from the databases (Fig. 1). We included one representative strain from *E. albertii*,* E. fergusonii* and each of eight *E. coli* phylogroups in this analysis. This analysis reconfirmed that the C-I strains formed a monophyletic sister lineage of *E. coli ss*. The ANI was over 98.3% within the C-I lineage and over 95.9% between the C-I strains and the eight representative *E. coli* strains*.* The C-II (“Ew”), Er (C-III/IV), Em (C-V), C-VI and C-VIII strains also formed a monophyletic lineage, respectively, with Er (C-IV) and Em (C-V) strains representing sister lineages.

More than half (59.0%) of the C-I strains were isolated in North America, while the others were isolated in various countries on the other 5 continents (Fig. 1 and Additional file 1: Fig. S3a). The majority of the C-I strains (77.3%) were isolated from livestock animals or humans (Additional file 1: Fig. S3b). In in silico serotyping, 99% of the C-I strains were serotyped as known *E. coli* O or H genotypes (Additional file 1: Fig. S3c). They were classified into 51 different O:H genotypes, among which the highest numbers of strains belonged to OG9:H45 (15%) or ONT:H45 (15%).

In contrast, more than half (51.5% to 95.5%) of the C-II (“Ew”), Er (C-III/IV) and Em (C-V) strains were from aquatic environments or wild animals (Fig. 1 and Additional file 1: Fig. S3b). In in silico serotyping, 38, 54 and 59% of the C-II (“Ew”), Er (C-III) and Er (C-IV) strains, respectively, were untypeable for both *E. coli* O and H genotypes (Additional file 1: Fig. S3c). While all the Em (C-V) strains were serotyped as known O or H genotypes, it is noteworthy that 99% were H56 with various O genotypes, with a high proportion of strains (52%) showing the ONT:H56 genotype.

### Distribution of known *E. coli* virulence genes in C-I strains

**Fig. 2:**
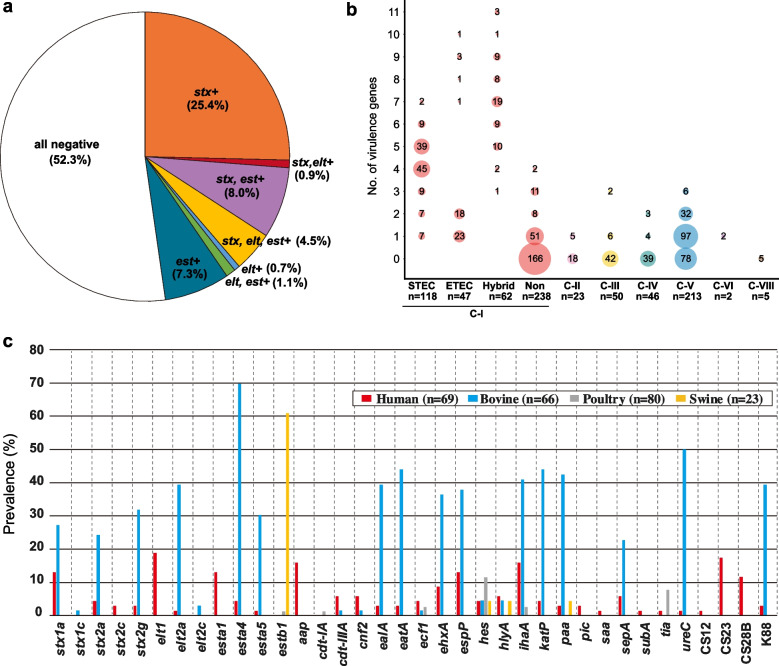
Distribution of virulence genes in C-I, other cryptic clades and recently defined *Escherichia* species. **a** Pie chart showing the distribution of the STEC and ETEC hallmark virulence genes in C-I strains. **b** Grid charts showing the numbers of virulence genes other than the hallmark virulence genes identified in C-I, other cryptic clades and recently defined species. C-I strains were grouped into four pathotypes. STEC: STEC-type, ETEC: ETEC-type, Hybrid: STEC/ETEC hybrid-type, Non: nontypeable. **c** Distribution of virulence genes in C-I strains from humans and livestock animals (bovine, poultry and swine)

We next analysed the distribution of the abovementioned hallmark virulence genes of the five major pathotypes of intestinal pathogenic *E. coli* in C-I strains (Figs. 1 and 2a and Additional file 1: Fig. S4). Although *eaeA*, *aggR* and *ipaH* were not detected in any of the C-I strains analysed here, many of the C-I strains (227 strains; accounting for nearly half of the strains analysed) were found to carry at least one of the *stx, elt* and *est* genes (Fig. 2a), in sharp contrast to C-II (“Ew”), Er (C-III/IV), Em (C-V), C-VI and C-VIII, in which only three and four strains of Em (C-V) were positive for *eaeA* and *ipaH*, respectively (Fig. 1). Although various subtypes of the three hallmark virulence genes were identified, the 227 C-I strains were classified into three types according to the profiles of these hallmark virulence genes: STEC (*n* = 118), ETEC (*n* = 47) and STEC/ETEC hybrid (*n* = 62) types.

The presence of STEC/ETEC hybrid-type C-I strains carrying *stx* and *est* was previously reported [1, 21]. In this study, STEC/ETEC hybrid-type C-I strains carrying various combinations of *stx*, *elt* and *est* were identified (Fig. 2a). Notably, in the *stx*-positive C-I strains (STEC- and STEC/ETEC hybrid types), *stx2a* was most frequently detected among the various subtypes of *stx1* and *stx2* genes (86 of 180 strains; Additional file 1: Fig. S4). In human and animal ETEC strains, various colonization factors (CFs) have been identified [27, 28]. Although, in the ETEC- and STEC/ETEC hybrid-type C-I strains (*est-* and/or *elt*-positive; *n* = 109), known ETEC colonization factors (CFs) were detected in only 17 strains, two types of K88-like CFs were identified in 41 C-I strains (Additional file 2: Table S1 and see Additional file 3: Supplementary text for details) [21, 27–33].

Various other known *E. coli* virulence genes were detected in the C-I strains (Fig. 1). Among the genes analysed, *espP* (encoding serine protease), *ehxA* (encoding enterohaemolysin) and *ihaA* (encoding adhesin) were most frequently detected (23.9 ~ 29.5% of the total C-I strains, Additional file 1: Fig. S4). *espP* and *ehxA* were identified on the virulence plasmids of STEC [34], and *ihaA* was identified on chromosomal integrative elements in STEC [35]. Consistent with these genomic locations, the distribution of these three genes was biased towards *stx*-positive strains (STEC- and STEC/ETEC hybrid-type strains) (Additional file 1: Fig. S5). In contrast, the distribution of *ealA, eatA, katP* and *paa*, which encode a pertussis toxin-like toxin, a serine protease, a catalase and an adhesin, respectively, was biased towards the *est-* and/or *elt*-positive strains (ETEC- and STEC/ETEC hybrid-type strains) (Additional file 1: Fig. S5)*.* Despite these biased distributions, various known *E. coli* virulence genes were apparently accumulated in C-I strains of the STEC and STEC/ETEC hybrid types (Fig. 2b), showing a clear contrast to the strains belonging to C-II (“Ew”), Er (C-III/IV) and Em (C-V). In these lineages, *cdt* subtypes (encoding cytolethal distending toxins), *hes* (haemagglutinin),* paa* (adhesin),* pic* (serine protease) and *sat* (autotransporter toxin) were rather frequently detected in Er (C-IV) or Em (C-V) (Fig. 1 and Additional file 1: Fig. S4).

As described above, most of the C-I strains were isolated from humans and livestock animals (especially bovines, poultry and swine) (Additional file 1: Fig. S3b). We thus analysed the difference in the distribution of 36 virulence genes identified in the C-I strains between different hosts (Fig. 2c). Although the difference in numbers of hosts should be considered, approximately half of the genes analysed (16 genes) were much more frequently detected in bovine isolates than in isolates from the other hosts. Among the STEC and ETEC hallmark virulence genes, the *stx1a, stx2a, stx2g, elt2a, esta4* and *esta5* genes were detected in both human and bovine isolates but more frequently in bovine isolates. In contrast, *elt1* and *esta1* were specifically distributed in human isolates. *estb1* was almost entirely specific to strains from swine.

**Fig. 3:**
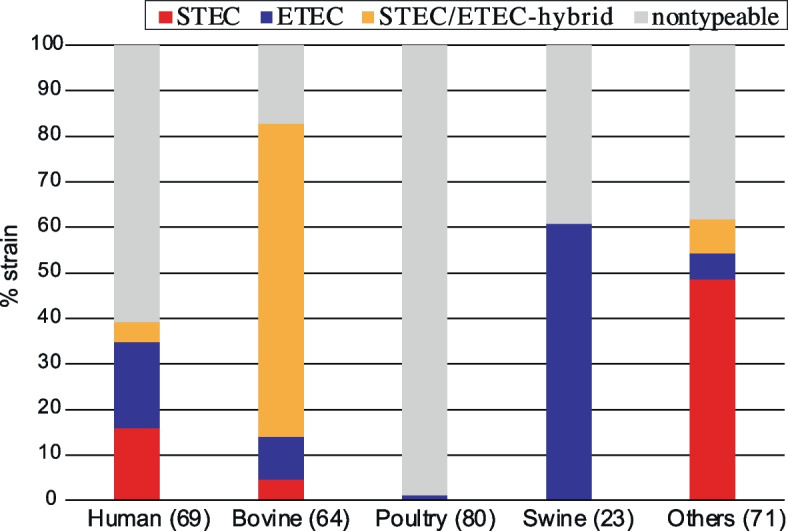
Distribution of STEC-, ETEC- and STEC/ETEC hybrid-type C-I strains in humans and livestock animals. “Others” includes wild animals (*n* = 12), companion animals (*n* = 4), food (*n* = 21), the environment (*n* = 28) and aquatic environments (*n* = 6)

Consistent with the distribution of the STEC and ETEC hallmark virulence genes in humans and livestock animals, more than 80% of the bovine isolates were classified into STEC-, ETEC- or STEC/ETEC hybrid-type strains, and STEC- and STEC/ETEC hybrid-type strains (i.e. *stx*-positive strains) were found in only human and bovine isolates (Fig. 3). In contrast, more than half of the swine isolates (14/23) were classified as ETEC-type strains, and only one poultry isolate (1/78) was classified as an ETEC-type strain (carrying *estb1*).

### Distribution of AMR genes in clade I

**Fig. 4:**
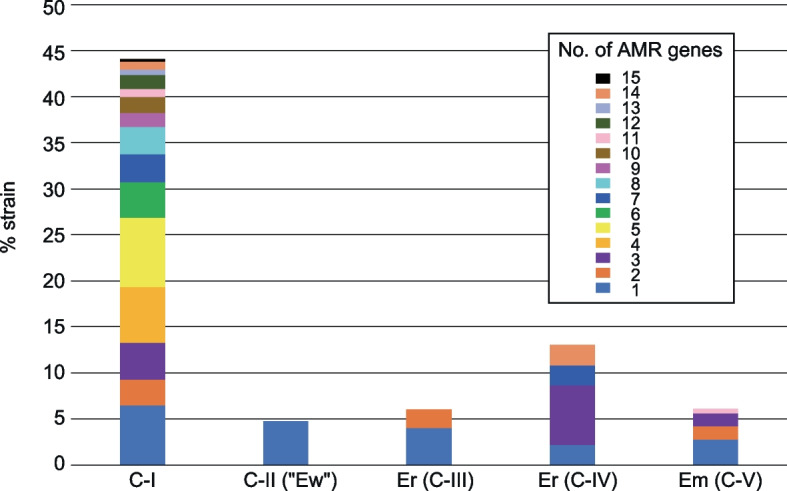
Prevalence of strains carrying AMR genes in C-I, other cryptic clades and recently defined *Escherichia* species. Bar charts indicate the percentage of strains carrying different numbers of AMR genes in each group of strains

Analysis of the distribution of horizontally acquired AMR genes revealed that more than 40% of the C-I strains (205 out of 465) carried at least one AMR gene, and most of these strains (175 out of 205) carried multiple AMR genes (up to 15 genes), again showing a clear difference from the C-II (“Ew”), Er (C-III/C-IV) and Em (C-V) strains (Fig. 4 and Additional file 2: Table S1). The number of C-I strains carrying AMR genes for more than three antibiotic classes was 153 (32.9%), while no such multidrug-resistant (MDR) strains were present in C-II (“Ew”) and Er (C-III), and only 4 (6.5%) and 3 (1.4%) strains of Er (C-IV) and Em (C-V), respectively, were MDR strains (Additional file 2: Table S1). In the C-I strains, a total of 79 AMR genes for 11 antibiotic classes were detected, among which *strA, strB, bla*_TEM-105_, *sul2* and *tetA* were widely distributed in C-I and detected in 94 (20.2%), 92 (19.8%), 78 (16.8%), 103 (22.2%) and 110 strains (23.7%), respectively (Additional file 2: Table S1 and Additional file 1: Figs. S6 and S7). Although several C-I sublineages exhibited low AMR gene prevalence, closely related strains did not always have the same AMR gene profile, suggesting repeated acquisitions of AMR genes in the C-I lineage.

Among the C-I strains from humans and livestock animals, the distribution patterns of AMR genes showed notable differences between hosts (Additional file 1: Fig. S8), which may be linked to the difference in the usage of antimicrobials. In particular, genes for metallo-β-lactamase and two other carbapenemases were detected only in human isolates, and most extended-spectrum β-lactamase (ESBL) genes (9/10) were detected in human and/or poultry isolates. These strains have been isolated since 2011 (Additional file 2: Table S1).

A more striking difference in the distribution pattern of AMR genes was observed between intestinal pathogenic strains (STEC-, ETEC- or STEC/ETEC hybrid-type strains) and the other strains (Additional file 1: Fig. S9). Strains lacking any of the STEC/ETEC hallmark virulence genes (nonintestinal pathogenic strains) contained more AMR genes than intestinal pathogenic strains; the proportion of strains containing no AMR genes was significantly lower in nonintestinal pathogenic strains (38%) than in the STEC- (86.4%; *P* = 2.2E − 16) and STEC/ETEC hybrid-type strains (72.6%; *P* = 9.5E − 07). Conversely, the average number of AMR genes carried by nonintestinal pathogenic strains (3.55 genes) was significantly higher than those in STEC- (0.53 genes; *P* = 6.7E − 15), ETEC- (2.26 genes; *P* = 0.033) and STEC/ETEC hybrid-type strains (1.40 genes; *P* = 9.3E − 05). The abovementioned genes for carbapenemases and ESBLs were also more frequently detected in nonintestinal pathogenic strains than in the other strains (Additional file 1: Fig. S9).

### MGEs carrying the STEC and ETEC hallmark virulence genes and AMR genes in C-I

Detailed analyses of MGEs are required to understand the mechanisms underlying the acquisition of virulence and AMR genes, but draft sequences do not provide enough information for such analyses. However, only four completely sequenced C-I genomes were available in the public databases (Additional file 2: Table S2). Moreover, among the STEC and ETEC hallmark virulence genes, only *stx1* was identified in two (89–3506 and 2013C-4282) of these completely sequenced genomes (Additional file 2: Table S1). Among virulence plasmids, the complete sequence of an *est*-encoding plasmid, p7v, was the only one available [23]. Therefore, we determined the complete genome sequences of three strains, namely, a human clinical isolate (10290) and two bovine isolates (KS-P079 and HH-P024) (Additional file 2: Table S2). These strains were selected as they belonged to C-I sublineages different from those of the abovementioned four strains and carried different combinations of STEC and ETEC hallmark virulence genes, including *stx2a*, *stx2g, esta4, esta5,* e*lt2a* and *elt2c* (Fig. 1 and Additional file 2: Table S1).

**Fig. 5:**
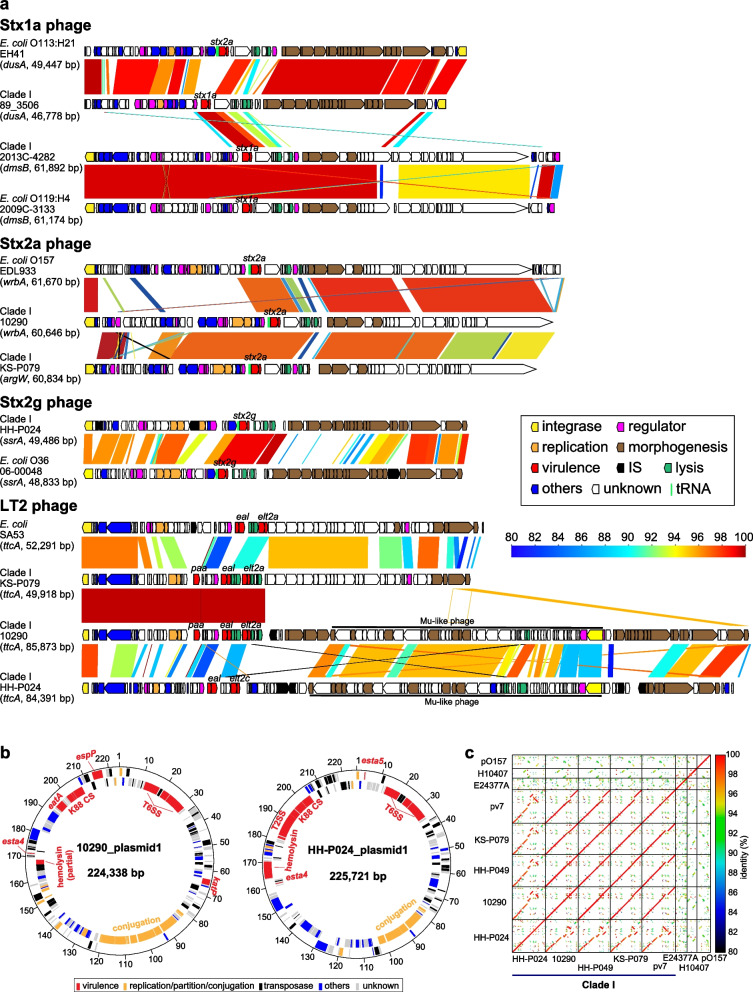
Genomic structures of MGEs carrying major virulence genes. **a** Genomic structures of Stx phages and LT2 phages. The genome structures of Stx2a, Stx2g and LT2 phages of the C-I strains whose complete genome sequences were determined are drawn to scale. Phages of *E. coli* strains are also shown for comparison. Homologous regions are indicated by shading, and sequence identities are indicated by different colours. Integration sites and sequence lengths of each phage are also indicated. **b** Circular maps of the virulence plasmids in the C-I strains 10,290 and HH-P024. **c** Dot-plot matrices of the completed C-I virulence plasmid sequences. Virulence plasmids of ETEC strains (E24377A and H10407) and a STEC O157 strain (Sakai) are also included for comparison

Similar to STEC strains, the *stx* genes were encoded by prophages in all five *stx*-positive C-I strains (Fig. 5a). The Stx phages of the C-I strains exhibited structural similarities to those of STEC strains that are available in publicly databases (see Additional file 3: Supplementary text for details and Additional file 2: Table S3).

The *elt2* genes in all three *elt2*-positive C-I strains were also encoded by prophages that were all integrated into *ttcA* (Fig. 5a and see Additional file 3: Supplementary text for details). A notable similarity between the C-I LT2 phages was the presence of *ealAB* genes encoding an ADP-ribosyltransferase toxin family, which was first identified on the LT2 phage of the ETEC strain SA53 [30]. The LT2a phages of two strains (KS-P079 and 10,290) additionally encoded Paa, which is involved in the early step of adhesion to intestinal epithelial cells [36, 37]. The genetic link of these two virulence-related genes with *elt2* explains, at least partly, the high prevalence of these two genes in ETEC- and STEC/ETEC hybrid-type C-I strains (Additional file 1: Fig. S5).

A large plasmid in three *est*-positive C-I strains (10290, KS-P079 and HH-P024) exhibited remarkable similarity to p7v (Fig. 5b, c and see Additional file 3: Supplemental text for details). The *esta4* gene, along with the genes for a K88 fimbria-like CF (Additional file 1: Fig. S10 and see Additional file 3: Supplemental text for details) and a type 6 secretion system (T6SS), was encoded by these C-I virulence plasmids (Fig. 5b). The plasmid in 10290/KS-P079/7v additionally encoded the genes for the EspP serine protease, KatP catalase, EatA serine protease, a type 2 secretion system (T2SS) and a partial enterohaemolysin (Ehx). The plasmid in HH-P024 also additionally encoded *esta5* and full-length genes for Ehx. Despite these differences, these virulence plasmids of the C-I strains exhibited chimeric features of STEC and ETEC virulence plasmids as previously reported [21]; EspP, KatP, Ehx and T2SS are associated with STEC virulence plasmids, and STs, EatA and the K88 fimbria-like CF are associated with ETEC virulence plasmids. The presence of the STEC/ETEC chimeric plasmids explains the high prevalence of these virulence genes in STEC/ETEC hybrid-type C-I strains (Additional file 1: Fig. S5). However, the backbones of these C-I virulence plasmids completely differed from those of the typical ETEC strains E23487A and H10407 and the typical STEC O157 strain Sakai (Fig. 5c).

Strain 10290 contained an additional large plasmid encoding multiple AMR genes (Additional file 1: Fig. S11). All AMR genes identified in this strain were encoded by this plasmid. The *sepA* gene encoding Shigella extracellular protein A, which is involved in tissue invasion [38], was also found in this plasmid.

### Production levels of Stx2 in *stx2*-positive C-I strains

**Fig. 6:**
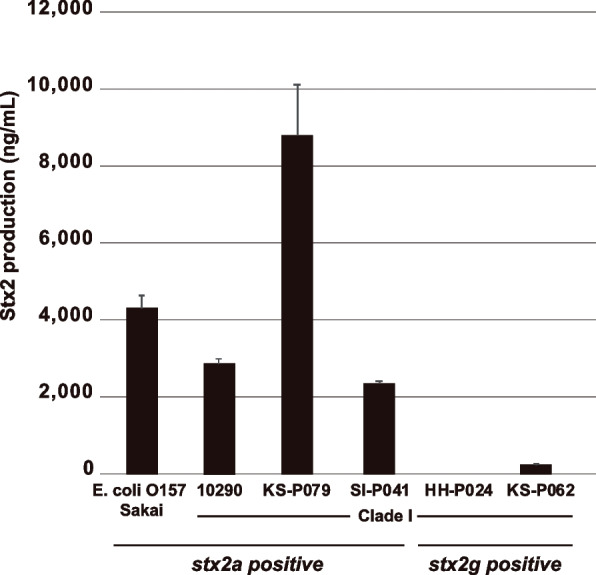
Stx production levels of C-I strains. Stx concentrations of five *stx2a*- and two *stx2g*-positive C-I strains after mitomycin treatment are shown. The STEC strain Sakai carrying *stx2a* is also included for comparison. The mean values of three measurements are presented. Error bars represent standard errors

A notable number of C-I strains (135 out of 465) were *stx2* positive, and Stx2 production is one of the known risk factors for severe STEC infections [39–41]. However, it is also known that mitomycin C (MMC)-induced Stx2 production levels vary significantly among STEC strains [32, 39–46]. Therefore, we analysed the Stx2 production levels of five *stx2*-positive C-I strains belonging to different C-I sublineages (Fig. 1); three were *stx2a* positive, and two were *stx2g* positive. The three *stx2a*-positive strains, including the human clinical isolate 10,290, exhibited high levels of Stx2 production more or less comparable to that of the STEC O157:H7 strain Sakai, which caused a large outbreak in 1996 in Sakai, Japan [35] (Fig. 6). In particular, the level in strain KS-P079 was two times higher than that in strain Sakai. In contrast, of the two *stx2g*-positive strains, the Stx2 production level in one strain was below the detection limit, and that in the other strain was much lower than those in the *stx2a*-positive strains.

## Discussion

C-I strains producing LT and ST have been isolated from children with diarrhoea, and thus C-I includes at least ETEC-type human pathogens [2]. However, the precise incidence and types of C-I infections remain unclear. This is partly due to the difficulty in distinguishing C-I strains from strains belonging to *E. coli ss*. In fact, most of the C-I genomes we identified in public databases (366 of 391 NCBI Biosample record-available genomes) were deposited as *E. coli* genomes. The PCR-based C-I detection system that we developed based on the genomic information of these C-I strains solved this problem, as shown in our retrospective surveys of C-I infections among the strains previously identified as *E. coli* from various sources. Although only two C-I strains were identified from clinical strains in this survey, it is notable that one of the two strains carried *stx2a*, *elt2a* and *esta4* and apparently caused a severe intestinal infection with bloody diarrhoea, as there is no report of apparent symptomatic human infection by Stx-producing C-I.

Our genomic analysis of the global C-I strain set including the C-I strains identified in this study along with strains belonging to other cryptic *Escherichia* clades and recently defined *Escherichia* species confirmed the phylogenetic position of C-I as a sister lineage of *E. coli* ss at the subspecies level (> 95% ANI) (Fig. 1). Moreover, our analysis revealed several important findings on the features of the C-I lineage that were not observed in other cryptic clades and recently defined *Escherichia* species. Among these, the most important finding was the marked accumulation of *E. coli* virulence genes in C-I, in particular the presence of STEC and/or ETEC hallmark virulence genes in half of the C-I strains in the dataset (Fig. 2a). These strains were classified into three pathotypes, namely, the STEC, ETEC and STEC/ETEC hybrid types, and all the types included notable numbers of human isolates (Fig. 3). This finding, together with the abovementioned cases of human infections caused by ETEC- and STEC/ETEC hybrid-type strains, indicates that a considerable portion of C-I strains are human pathogens.

In this regard, it is noteworthy that among the various subtypes of *stx* detected in *stx*-positive C-I strains (180 of 465 strains), the most prevalent was *stx2a* (86 strains), followed by *stx1a* (62 strains) (Additional file 1: Fig. S4). STEC strains carrying *stx1a* and/or *stx2a* are often associated with human diseases [46], and *stx2a*-positive strains are most frequently associated with severe diseases, including haemolytic colitis and haemolytic uraemic syndrome [39–41]. In addition, although it is known that STEC strains produce various levels of Stx2 [32, 42–45], which is associated with the variation in the Stx2 phage genome [32], the levels of Stx2 production and the genomic structures of Stx2a phages in the *stx2a*-positive C-I strains analysed were comparable to those of a representative STEC O157 strain (Figs. 5 and 6). In contrast, as reported in STEC [47–49], the Stx2 production levels of C-I strains carrying only *stx2g*, which was also the *stx2* subtype frequently detected in the strain set (33 strains; Additional file 1: Fig. S4), were much lower than those in the *stx2a*-positive strains or were below the detection limit (Fig. 6). Moreover, all the *stx2g*-positive strains identified possessed *esta4* and/or *esta5* (Additional file 2: Table S1), and *stx2g*-positive STEC strains are rarely isolated from humans [50, 51]. Therefore, it remains unclear to what extent *stx2g* is responsible for human diseases.

Regarding the pathogenicity of C-I strains, it is also noteworthy that among the three subtypes of *elt* (*elt1*, *elt2a* and *elt2c*) found in 54 *elt*-positive C-I strains (Additional file 1: Fig. S4 and Additional file 2: Table S1), *elt1*, which is predominantly associated with human disease, was detected in 22 strains, and *elt2* (*elt2a* and *elt2c*), which is mainly associated with diseases in livestock animals [52], was detected in 32 strains. Moreover, all *elt1*-positive strains were human isolates, and almost all the *elt2*-positive strains (28/29) were bovine isolates (Fig. 2c). ST is also divided into two types: STa is mainly responsible for human diseases, and STb is associated with diseases in livestock animals, particularly piglets and calves [52]. The *est* genes detected in 97 C-I strains (*esta1*, *esta4, esta5* and *estb1*) also exhibited host-specific distributions; e*sta1* was specifically detected in human isolates (*n* = 17), and *esta4,* e*sta5* and e*stb1* showed highly biased distributions in bovine and swine isolates (*n* = 61, 28 and 17, respectively) (Fig. 2c). The distribution patterns of CFs were also apparently host specific (Fig. 2c). These data suggest that C-I is also a source of LT- and/or ST-producing strains pathogenic to human and livestock animals, although the roles of *esta4* and *esta5* as virulence factors are unknown and disease information for these *elt*/*est-*positive livestock animal isolates is not available.

In terms of the potential virulence of C-I strains, it is also noteworthy that the other potential virulence factors of ETEC and STEC were accumulated in intestinal pathogenic C-I strains (Fig. 2b and Additional file 1: Fig. S4). Although the roles of these factors in pathogenesis have not been fully understood even in STEC and ETEC, many of them are genetically linked with MGEs that were found to be associated with ETEC and STEC. A more important finding related to potential sources of human infection caused by these intestinal pathogenic C-I strains is that most of the STEC- and STEC/ETEC hybrid-type strains were bovine isolates, accounting for 77% of the bovine isolates (Fig. 3). This finding suggests that bovines are the major reservoir of STEC- and STEC/ETEC hybrid-type C-I strains and that the source of human infections caused by these strains is similar to that of STEC [53–55]. In fact, beef was the most likely infection source in the human case of Stx2a-producing C-I strain infection identified in this study.

In addition to *stx, elt* and *est*, various virulence genes were more frequently detected in C-I strains isolated from bovines compare to those isolated from humans and the other animals (Fig. 2c). Previous research has indicated that among bovine commensal *E. coli* strains, various virulence genes are accumulated in *stx*-positive strains, which implies that these virulence factors are functionally associated with Stx and likely confer benefits to these *E. coli* strains as they adapt to the bovine intestinal environment. This phenomenon may also hold true for C-I strains.

It is also of note that although the known *E. coli* virulence factors were rarely identified in recently defined *Escherichia* species and other cryptic clades, *cdt-*IV was identified in many C-V strains (82 out of 213). The *cdt* was initially discovered in *E. coli* and has been categorized into five subtypes (*cdt*-I to *cdt*-V) [56]. The *cdt* is only present in a subset of *E. coli* strains, while most *E. albertii* strains possess the *cdt* belonging to the *cdt*-II/III/V. As *E. albertii* strains are also frequently isolate from the environment and wild animals, *cdt* may be necessary to adapt these natural niches in *E. albertii* and C-V strains.

The accumulation of AMR genes is another important feature of C-I. Many strains possess multiple AMR genes (Fig. 4 and Additional file 1: Figs. S6 and S7), and as many as 32.9% of the C-I strains in our dataset were genotypically MDR stains. The AMR gene-positive strains were widely distributed in the C-I lineage irrespective of isolation source (Additional file 1: Figs. S7 and S8), but nonintestinal pathogenic strains carried more AMR genes (Additional file 1: Fig. S9). These findings suggest the close association of C-I strains, particularly nonintestinal pathogenic strains, with humans and livestock animals. Among the 55 AMR genes detected, the presence of genes for ESBLs and carbapenemases has particularly strong clinical implications considering their wide spectrum. Although the prevalence of such strains was not high, their distribution was apparently biased to human and poultry isolates (Additional file 1: Fig. S8). This may also support the close association of nonintestinal pathogenic C-I strains with humans, for which third- and fourth-generation cephalosporins and carbapenems are increasingly prescribed, although the background underlying the association of these AMR genes with poultry isolates is unclear.

The present study, however, has limitations that should be considered. The primary limitation of our study is the possibility of sampling bias resulting from the employment of a substantial proportion of publicly available genome data. Specifically, in the context of C-I strains, genomic sequencing may be biased towards strains that harbour virulence and AMR genes as well as those that isolated from clinical and veterinary sources, such as humans and livestock animals. This can be attributed to extensive surveillances and large-scale genomic studies of pathogenic *E. coli* strains. Another limitation of this study is that only a sporadic case of clinically relevant C-I isolate was identified, and no C-I isolate that causes disease in livestock animals was detected. Consequently, the severity and prevalence of C-I infections in humans and animals remain obscure. Nevertheless, despite these limitations, through analyses using a large number of strains that were precisely classified as C-I and other cryptic clades, our study has provided invaluable insights into the global population structure and virulence potential of C-I.

## Conclusions

*Escherichia* cryptic clade I (referred to as C-I in this article) includes human and animal intestinal pathogenic strains of STEC, ETEC or STEC/ETEC hybrid types, and the major source of human infections caused by STEC- and STEC/ETEC hybrid-type strains is most likely bovines. Multiple AMR genes have accumulated in many C-I strains, particularly nonintestinal pathogenic strains, suggesting their close association with humans and livestock animals. To better understand the features of C-I strains and their infections, extensive surveillance of C-I strains and larger population studies using strain sets well supplemented with metadata are needed. Although C-I strains are difficult to distinguish from *E. coli ss* strains by routine species identification methods, the PCR system highly specific to C-I developed in this study will be a powerful tool for screening and identifying C-I strains.

## Methods

### Database searches of the genome sequences of C-I strains

To identify strains belonging to C-I, C-II (“Ew”), Er (C-III/IV), Em (C-V) and other cryptic clades in the 170,489 *Escherichia* spp. genomes available in EnteroBase (last access: 2021/07/31), we excluded those deposited as *E. albertii* or *E. fergusonii* and those assigned to any of the *E. coli* phylogroups by both ClermonTyping and EzClermont, obtaining a total of 1088 genomes, including those of the type strains of Er and Em (strains OPT1704^T^ and HT073016^T^, respectively). To eliminate low-quality assembly, CheckM was employed to assess the completeness and contamination levels [57] (Additional file 2: Table S1). Genomes with ≥ 90% completeness have been categorized as nearly complete, whereas those with > 10% contamination have been classified as having a high degree of contamination [57]. In this study, we removed 23 genomes that exhibited either < 95% completeness or > 10% contamination. We then classified the remaining 1065 genomes into *Escherichia* species or cryptic clades by whole-genome-based phylogenomic analysis. Raw read data were also obtained from the Enterobase database if available. Additionally, the genome sequences of a representative strain for *E. fergusonii* and *E. albertii*, alongside each of the major *E. coli* phylogroupes, were obtained from EnteroBase and used as references [ATCC_35469T (*E. fergusonii*), EC06-170 (*E. albertii),* MG1655 (*E. coli* phylogroup A), SE11 (*E. coli* phylogroup B1), SE15 (*E. coli* phylogroup B1), APEC O78 (*E. coli* phylogroup C), UMN026 (*E. coli* phylogroup D), CB9615 (*E. coli* phylogroup E), SMS-3–5 (*E. coli* phylogroup F) and PSUO78 (*E. coli* phylogroup G)]. The genome sequences of 17 C-II strains [9] that were available in the NCBI database but not in the EnteroBase database were also obtained from the NCBI database. Detailed information for the strains used in this study is provided in Additional file 2: Table S1.

### SNP detection and phylogenomic analysis

To construct core gene-based phylogenomic trees, pangenome analyses of each strain set were performed using Roary with the options (-i 80 -cd 100 -s) [58]. SNP sites were extracted from the core gene alignment using snp-sites [59], and ML phylogenomic trees were constructed using RAxML [60] with the GTR-GAMMA model of nucleotide substitution and 500 bootstrap replicates and displayed and annotated using iTOL [61].

### Development of the PCR-based C-I detection system

Through a pangenome analysis of 792 genomes of C-I, C-II (“Ew”), Er (C-III/C-IV), Em (C-V), C-VI and C-VIII strains and the reference strains of *E. albertii, E. fergusonii* and *E. coli* using Roary [58] (-i 80 -cd 100 -s) and a subsequent pangenome-wide association study using Scoary [62], we identified one gene (1368 bp in length) that was conserved in nearly all C-I strains (452/453) and completely absent in the other strains analysed and designed a C-I-specific primer pair (F: TGCGAAACATGAGACAGTCC, R: TTCGGTAAGCCCATTCTCTG) for this gene. PCR was performed using the primer pairs and KAPATaq polymerase (Kapa Biosystems, Woburn, MA, USA) with a programme of 30 cycles of 20 s at 98 °C, 30 s at 60 °C and 1 min at 72 °C.

### Genome sequencing, assembly and annotation

Genomic DNAs of the C-I strains isolated in this study were purified from 1 ml of an overnight culture using the DNeasy Blood and Tissue Kit (Qiagen). Libraries were prepared using the Nextera XT DNA Sample Preparation Kit (Illumina, CA, USA) or the NEBNext Ultra DNA Library Prep Kit for Illumina (NEB) and sequenced on the Illumina MiSeq platform to generate 300-bp paired-end reads. Genome assembly of the Illumina sequence reads was performed using the Platanus_B assembler [63].

The four finished C-I genomes (HH-P024, KS-P079 and 10,290) were obtained by mixed assembly of MiSeq and MinION (Oxford Nanopore Technologies, Oxford, UK) reads using the Unicycler pipeline version 0.4.4 with the default settings [64]. Libraries for long-read sequencing were prepared using the Rapid Barcoding Kit (SQK-RBK004; Oxford Nanopore Technologies, Ltd.) and sequenced using an R9.4.1 flow cell on the Oxford Nanopore MinION platform. Annotation was carried out using the DDBJ Fast Annotation and Submission Tool (DFAST) with the default settings [65]. Comparisons of prophage, plasmid and CS locus sequences were conducted using GenomeMatcher version 3.02 [66].

### In silico serotyping and identification of virulence and AMR genes

In silico serotyping was conducted by using ECTyper version 1.0.0 with the default settings [67]. The identification and variant typing of *E. coli* hallmark virulence genes were performed using an in-house database (Additional file 2: Table S4) by a BLASTN search with a threshold of 99% identity and 90% coverage and a read mapping-based strategy by SRST2 [68] with the options “–max-divergence 1”. Genes detected by either the BLASTN or SRST2 analyses were considered to be present. The other *E. coli* virulence genes were identified by BLASTN searches using an in-house database (Additional file 2: Table S5) with a threshold of 90% identity and 90% coverage. The identification of genes encoding the known ETEC colonization factors was conducted via BLASTN searches of the scaffold sequences of each strain against the database file ETEC_vir_db (https://github.com/avonm/ETEC_vir_db). The result was considered positive when all genes of a given CF were conserved (> 90% identity and > 80% coverage). AMR genes were identified using ABRicate version 1.0.1 (https://github.com/tseemann/abricate) with the default settings using the CARD (http://www.mgc.ac.cn/VFs) databases.

### Stx2 production assay

Bacterial cells were inoculated into 40 ml of lysogeny broth and grown to mid-log phase at 37 °C with shaking. MMC was then added to the culture at a final concentration of 500 ng/ml. At 5 h after MMC addition, 100 µl of the culture was collected and immediately subjected to sonication using a Bioruptor (Cosmo Bio, Tokyo, Japan). The soluble fractions of each cell lysate were obtained via centrifugation at 14,000 × *g* at 4 °C for 10 min. The Stx2 concentration in each cell lysate was determined using a previously described sandwich ELISA [69] using RIDASCREEN Verotoxin microtiter plates (R-Biopharm GmbH, Germany) coated with capture antibodies that recognize Stx2 and monoclonal antibodies against Stx2 (LSBio, WA, USA) conjugated with horseradish peroxidase using a Peroxidase Labeling Kit–NH2 (Dojindo, Kumamoto, Japan) as detection antibodies. Note that both Stx2a and Stx2g could be quantified at a certain level of accuracy using the detection antibodies [70].

### Statistical analysis

All statistical analyses were performed using R version 3.6.1 (R Project for Statistical Computing). The prop test was conducted to examine the differences in the proportion of strains containing no AMR gene between nonintestinal pathogenic strains and intestinal pathogenic strains (STEC-, ETEC- or STEC/ETEC hybrid-type strains). The *t* test was used to examine the differences in the average number of AMR genes between strains.

## Supplementary Information

**Additional file 1:** **Figure S1.** Core gene-based phylogenomic tree of the strains in EnteroBase that were suspected to belong to *Escherichia* cryptic clades and recently defined *Escherichia* species. **Figure S2.** Agarose gel electrophoresis analysis of the products obtained by C-I detection PCR. **Figure S3.** Continents, sources and serotype distributions of strains belonging to C-I, other cryptic clades and recently defined *Escherichia* species. **Figure S4.** Summary of the prevalence of virulence genes in C-I, other cryptic clades and recently defined *Escherichia* species. **Figure S5.** Distribution of AMR genes in STEC-type, ETEC-type, STEC/ETEC hybrid-type and nonintestinal pathogenic C-I strains. **Figure S6.** Summary of the prevalence of AMR genes in C-I. **Figure S7.** Distribution of AMR genes in C-I, other cryptic clades and recently defined *Escherichia* species. **Figure S8.** Summary of the prevalence of AMR genes in C-I strains from humans and livestock animals. **Figure S9.** Distribution of AMR genes in STEC-type, ETEC-type, STEC/ETEC hybrid-type and nonintestinal pathogenic C-I strains. **Figure S10.** Comparison of genes in the K88-like CF loci with those in the K88 CS locus. **Figure S11.** Circular map of the plasmid encoding multiple AMR genes and the *sepA* gene in the C-I strain 10290.

**Additional file 2: Table S1.** Characteristics of strains used in this study. **Table S2.** Length of chromosome and plasmids in cryptic clade I strains in which the genome sequence was compldetely determined. **Table S3.** Alignment coverages of the completely sequenced C-I phages and plasmids encoding major virulence genes with the scaffold sequences of the C-I strains. **Table S4.** List of the nucleotide sequences of the major virulence genes used in this study. **Table S5.** List of the nucleotide sequences of *E. coli* virulence genes used in this study.

**Additional file 3:** **Supplementary Text.** Colonization factors in C-I strains and structural comparisons of the Stx and LT phages and the virulence plasmids in C-I strains.

## Data Availability

All sequence data generated in this study are available in the DDBJ/EMBL/GenBank BioProject under accession number PRJDB7924 [71].

## Abbreviations

STEC: Stx-producing *E. coli*
ETEC: Enterotoxigenic *E. coli*
EPEC: Enteropathogenic *E. coli*
EAEC: Enteroaggregative *E. coli*
EIEC: Enteroinvasive *E. coli*
ANI: Average nucleotide identity
MGE: Mobile genetic element
T2SS: Type 2 secretion system
T3SS: Type III secretion system
T6SS: Type 6 secretion system
LT: Heat-labile enterotoxin
ST: Heat-stable enterotoxin
AMR: Antimicrobial resistance
CF: Colonization factors
ESBL: Extended-spectrum β-lactamase

## References

[1] Gangiredla J, Mammel MK, Barnaba TJ, Tartera C, Gebru ST, Patel IR (2018). Draft genome sequences of *Escherichia albertii, Escherichia fergusonii,* and strains belonging to six cryptic lineages of *Escherichia* spp. Genome Announc.

[2] Walk ST. The “Cryptic” *Escherichia*. EcoSal Plus. 2015;6(2). https://journals.asm.org/doi/10.1128/ecosalplus.ESP-0002-2015. 10.1128/ecosalplus.esp-0002-2015 PMC11575852 26435255

[3] Walk ST, Alm EW, Gordon DM, Ram JL, Toranzos GA, Tiedje JM (2009). Cryptic lineages of the genus *Escherichia*. Appl Environ Microbiol.

[4] van der Putten  BCL, Matamoros  S, Mende DR, Scholl ER, Schultsz  C, Consortium C (2021). *Escherichia ruysiae* sp. nov., a novel Gram-stain-negative bacterium, isolated from a faecal sample of an international traveller. Int J Syst Evol Microbiol.

[5] Liu S, Jin D, Lan R, Wang Y, Meng Q, Dai H (2015). *Escherichia marmotae *sp. nov., isolated from faeces of *Marmota himalayana*. Int J Syst Evol Microbiol..

[6] Gilroy R, Ravi A, Getino M, Pursley I, Horton DL, Alikhan NF (2021). Extensive microbial diversity within the chicken gut microbiome revealed by metagenomics and culture. PeerJ.

[7] Clermont O, Gordon DM, Brisse S, Walk ST, Denamur E (2011). Characterization of the cryptic *Escherichia* lineages: rapid identification and prevalence. Environ Microbiol.

[8] Luo C, Walk ST, Gordon DM, Feldgarden M, Tiedje JM, Konstantinidis KT (2011). Genome sequencing of environmental *Escherichia coli* expands understanding of the ecology and speciation of the model bacterial species. Proc Natl Acad Sci U S A.

[9] Shen Z, Koh XP, Yu Y, Woo CF, Tong Y, Lau SCK. Draft genome sequences of 16 strains of *Escherichia* Cryptic Clade II isolated from intertidal sediment in Hong Kong. Microbiol Resour Announc. 2019;8(29):e00416-19. 10.1128/MRA.00416-19 PMC6639607 31320428

[10] Chun J, Oren A, Ventosa A, Christensen H, Arahal DR, da Costa MS (2018). Proposed minimal standards for the use of genome data for the taxonomy of prokaryotes. Int J Syst Evol Microbiol.

[11] Goris J, Konstantinidis KT, Klappenbach JA, Coenye T, Vandamme P, Tiedje JM (2007). DNA-DNA hybridization values and their relationship to whole-genome sequence similarities. Int J Syst Evol Microbiol.

[12] Achtman M, Zhou Z, Charlesworth J, Baxter L (1861). EnteroBase: hierarchical clustering of 100 000s of bacterial genomes into species/subspecies and populations. Philos Trans R Soc Lond B Biol Sci.

[13] Brenner  DJ, Davis BR, Steigerwalt AG, Riddle CF, McWhorter AC, Allen SD (1982). Atypical biogroups of *Escherichia coli* found in clinical specimens and description of *Escherichia hermannii* sp. nov. J Clin Microbiol.

[14] Hata  H, Natori T, Mizuno  T, Kanazawa I, Eldesouky I, Hayashi M (2016). Phylogenetics of family *Enterobacteriaceae* and proposal to reclassify *Escherichia hermannii* and *Salmonella subterranea* as *Atlantibacter hermannii* and *Atlantibacter subterranea* gen. nov., comb. nov.. Microbiol Immunol.

[15] Kaper JB, Nataro JP, Mobley HL (2004). Pathogenic *Escherichia coli*. Nat Rev Microbiol.

[16] Gaastra W, Kusters JG, van Duijkeren E, Lipman LJA (2014). Escherichia fergusonii. Vet Microbiol.

[17] Inglis TJJ, Merritt AJ, Bzdyl N, Lansley S, Urosevic MN (2015). First bacteraemic human infection with *Escherichia albertii*. New Microbes New Infect.

[18] Ooka T, Seto K, Kawano K, Kobayashi H, Etoh Y, Ichihara S (2012). Clinical significance of *Escherichia albertii*. Emerg Infect Dis.

[19] Ooka T, Tokuoka E, Furukawa M, Nagamura T, Ogura Y, Arisawa K (2013). Human gastroenteritis outbreak associated with *Escherichia albertii*. Japan Emerg Infect Dis.

[20] Leung PH, Peiris JS, Ng WW, Robins-Browne RM, Bettelheim KA, Yam WC (2003). A newly discovered verotoxin variant, VT2g, produced by bovine verocytotoxigenic *Escherichia coli*. Appl Environ Microbiol.

[21] Leonard SR, Mammel MK, Rasko DA, Lacher DW (2016). Hybrid Shiga toxin-producing and enterotoxigenic *Escherichia* sp. Cryptic Lineage 1 Strain 7v Harbors a Hybrid Plasmid. Appl Environ Microbiol.

[22] Steinsland H, Lacher DW, Sommerfelt H, Whittam TS (2010). Ancestral lineages of human enterotoxigenic *Escherichia coli*. J Clin Microbiol.

[23] Zhou Z, Alikhan NF, Mohamed K, Fan Y, Agama Study G, Achtman M (2020). The EnteroBase user's guide, with case studies on *Salmonella* transmissions, *Yersinia pestis* phylogeny, and *Escherichia* core genomic diversity. Genome Res.

[24] Beghain J, Bridier-Nahmias A, Le Nagard H, Denamur E, Clermont O (2018). ClermonTyping: an easy-to-use and accurate in silico method for *Escherichia* genus strain phylotyping. Microb Genom.

[25] Waters NR, Abram F, Brennan F, Holmes A, Pritchard L (2020). Easy phylotyping of *Escherichia coli* via the EzClermont web app and command-line tool. Access Microbiol.

[26] Arimizu Y, Kirino Y, Sato MP, Uno K, Sato T, Gotoh Y (2019). Large-scale genome analysis of bovine commensal *Escherichia coli* reveals that bovine-adapted *E. coli* lineages are serving as evolutionary sources of the emergence of human intestinal pathogenic strains. Genome Res.

[27] von Mentzer A, Connor TR, Wieler LH, Semmler T, Iguchi A, Thomson NR (2014). Identification of enterotoxigenic *Escherichia coli* (ETEC) clades with long-term global distribution. Nat Genet.

[28] Dubreuil JD, Isaacson RE, Schifferli DM. Animal Enterotoxigenic *Escherichia coli*. EcoSal Plus. 2016;7(1). https://journals.asm.org/doi/abs/10.1128/ecosalplus.ESP-0006-2016. 10.1128/ecosalplus.esp-0006-2016 PMC5123703 27735786

[29] Allue-Guardia A, Koenig SSK, Martinez RA, Rodriguez AL, Bosilevac JM, Feng P (2022). Pathogenomes and variations in Shiga toxin production among geographically distinct clones of *Escherichia coli* O113:H21. Microb Genom.

[30] Jobling MG (2016). The chromosomal nature of LT-II enterotoxins solved: a lambdoid prophage encodes both LT-II and one of two novel pertussis-toxin-like toxin family members in type II enterotoxigenic *Escherichia coli*. Pathog Dis.

[31] Lindsey RL, Knipe K, Rowe L, Garcia-Toledo L, Loparev V, Juieng P (2015). Complete genome sequences of two Shiga toxin-producing *Escherichia coli* strains from serotypes O119:H4 and O165:H25. Genome Announc.

[32] Ogura Y, Mondal SI, Islam MR, Mako T, Arisawa K, Katsura K (2015). The Shiga toxin 2 production level in enterohemorrhagic *Escherichia coli* O157:H7 is correlated with the subtypes of toxin-encoding phage. Sci Rep.

[33] Wells JG, Davis BR, Wachsmuth IK, Riley LW, Remis RS, Sokolow R (1983). Laboratory investigation of hemorrhagic colitis outbreaks associated with a rare *Escherichia coli* serotype. J Clin Microbiol.

[34] Makino K, Ishii K, Yasunaga T, Hattori M, Yokoyama K, Yutsudo CH (1998). Complete nucleotide sequences of 93-kb and 3.3-kb plasmids of an enterohemorrhagic *Escherichia coli* O157:H7 derived from Sakai outbreak. DNA Res..

[35] Hayashi T, Makino K, Ohnishi M, Kurokawa K, Ishii K, Yokoyama K (2001). Complete genome sequence of enterohemorrhagic *Escherichia coli* O157:H7 and genomic comparison with a laboratory strain K-12. DNA Res.

[36] An H, Fairbrother JM, Desautels C, Harel J (1999). Distribution of a novel locus called Paa (porcine attaching and effacing associated) among enteric *Escherichia coli*. Adv Exp Med Biol.

[37] Batisson I, Guimond MP, Girard F, An H, Zhu C, Oswald E (2003). Characterization of the novel factor *paa* involved in the early steps of the adhesion mechanism of attaching and effacing *Escherichia coli*. Infect Immun.

[38] Benjelloun-Touimi Z, Sansonetti PJ, Parsot C (1995). SepA, the major extracellular protein of *Shigella flexneri*: autonomous secretion and involvement in tissue invasion. Mol Microbiol.

[39] Bielaszewska M, Mellmann A, Bletz S, Zhang W, Kock R, Kossow A (2013). Enterohemorrhagic *Escherichia coli* O26:H11/H-: a new virulent clone emerges in Europe. Clin Infect Dis.

[40] Dallman TJ, Ashton PM, Byrne L, Perry NT, Petrovska L, Ellis R (2015). Applying phylogenomics to understand the emergence of Shiga-toxin-producing *Escherichia coli* O157:H7 strains causing severe human disease in the UK. Microb Genom.

[41] Persson S, Olsen KE, Ethelberg S, Scheutz F (2007). Subtyping method for *Escherichia coli* shiga toxin (verocytotoxin) 2 variants and correlations to clinical manifestations. J Clin Microbiol.

[42] de Sablet T, Bertin Y, Vareille M, Girardeau JP, Garrivier A, Gobert AP (2008). Differential expression of *stx2* variants in Shiga toxin-producing *Escherichia coli* belonging to seropathotypes A and C. Microbiology (Reading).

[43] Kawano K, Okada M, Haga T, Maeda K, Goto Y (2008). Relationship between pathogenicity for humans and *stx* genotype in Shiga toxin-producing *Escherichia coli* serotype O157. Eur J Clin Microbiol Infect Dis.

[44] Kulasekara BR, Jacobs M, Zhou Y, Wu Z, Sims E, Saenphimmachak C (2009). Analysis of the genome of the *Escherichia coli* O157:H7 2006 spinach-associated outbreak isolate indicates candidate genes that may enhance virulence. Infect Immun.

[45] Zangari T, Melton-Celsa AR, Panda A, Smith MA, Tatarov I, De Tolla L (2014). Enhanced virulence of the *Escherichia coli* O157:H7 spinach-associated outbreak strain in two animal models is associated with higher levels of Stx2 production after induction with ciprofloxacin. Infect Immun.

[46] Melton-Celsa AR (2014). Shiga Toxin (Stx) Classification, structure, and function. Microbiol Spectr.

[47] Bai X, Zhang J, Ambikan A, Jernberg C, Ehricht R, Scheutz F (2019). Molecular characterization and comparative genomics of clinical hybrid Shiga toxin-producing and enterotoxigenic *Escherichia coli* (STEC/ETEC) Strains in Sweden. Sci Rep.

[48] Granobles Velandia CV, Kruger A, Parma YR, Parma AE, Lucchesi PM (2012). Differences in Shiga toxin and phage production among stx(2g)-positive STEC strains. Front Cell Infect Microbiol.

[49] Prager R, Fruth A, Busch U, Tietze E (2011). Comparative analysis of virulence genes, genetic diversity, and phylogeny of Shiga toxin 2g and heat-stable enterotoxin STIa encoding *Escherichia coli* isolates from humans, animals, and environmental sources. Int J Med Microbiol.

[50] De Rauw K, Jacobs S, Pierard D (2018). Twenty-seven years of screening for Shiga toxin-producing *Escherichia coli* in a university hospital. Brussels, Belgium, 1987–2014. PLoS One.

[51] Melton-Celsa AR, O'Brien AD. New therapeutic developments against Shiga toxin-producing *Escherichia coli*. In: Environmental Microbial Forensics. Washington, DC: American Society for Microbiology; 2015. pp. 341–358.

[52] Mainil J (2013). *Escherichia coli* virulence factors. Vet Immunol Immunopathol.

[53] Beutin L, Geier D, Steinruck H, Zimmermann S, Scheutz F (1993). Prevalence and some properties of verotoxin (Shiga-like toxin)-producing *Escherichia coli* in seven different species of healthy domestic animals. J Clin Microbiol.

[54] Kolenda R, Burdukiewicz M, Schierack P (2015). A systematic review and meta-analysis of the epidemiology of pathogenic *Escherichia coli* of calves and the role of calves as reservoirs for human pathogenic *E. coli*. Front Cell Infect Microbiol..

[55] Dallman TJ, Jalava K, Verlander NQ, Gally D, Jenkins C, Godbole G (2022). Identification of domestic reservoirs and common exposures in an emerging lineage of Shiga toxin-producing *Escherichia coli* O157:H7 in England: a genomic epidemiological analysis. Lancet Microbe.

[56] Gomes TAT, Ooka T, Hernandes RT, Yamamoto D, Hayashi T. *Escherichia albertii* Pathogenesis. EcoSal Plus. 2020;9(1). https://journals.asm.org/doi/10.1128/ecosalplus.ESP-0015-2019. 10.1128/ecosalplus.esp-0015-2019 PMC11168576 32588811

[57] Parks DH, Imelfort M, Skennerton CT, Hugenholtz P, Tyson GW (2015). CheckM: assessing the quality of microbial genomes recovered from isolates, single cells, and metagenomes. Genome Res.

[58] Page AJ, Cummins CA, Hunt M, Wong VK, Reuter S, Holden MT (2015). Roary: rapid large-scale prokaryote pan genome analysis. Bioinformatics.

[59] Page AJ, Taylor B, Delaney AJ, Soares J, Seemann T, Keane JA (2016). SNP-sites: rapid efficient extraction of SNPs from multi-FASTA alignments. Microb Genom.

[60] Stamatakis A (2006). RAxML-VI-HPC: maximum likelihood-based phylogenetic analyses with thousands of taxa and mixed models. Bioinformatics.

[61] Letunic I, Bork P (2016). Interactive tree of life (iTOL) v3: an online tool for the display and annotation of phylogenetic and other trees. Nucleic Acids Res.

[62] Brynildsrud O, Bohlin J, Scheffer L, Eldholm V (2016). Rapid scoring of genes in microbial pan-genome-wide association studies with Scoary. Genome Biol.

[63] Kajitani R, Yoshimura D, Ogura Y, Gotoh Y, Hayashi T, Itoh T (2020). Platanus_B: an accurate de novo assembler for bacterial genomes using an iterative error-removal process. DNA Res.

[64] Wick RR, Judd LM, Gorrie CL, Holt KE (2017). Unicycler: Resolving bacterial genome assemblies from short and long sequencing reads. PLoS Comput Biol.

[65] Tanizawa Y, Fujisawa T, Nakamura Y (2018). DFAST: a flexible prokaryotic genome annotation pipeline for faster genome publication. Bioinformatics.

[66] Ohtsubo Y, Ikeda-Ohtsubo W, Nagata Y, Tsuda M (2008). GenomeMatcher: a graphical user interface for DNA sequence comparison. BMC Bioinformatics.

[67] Bessonov K, Laing C, Robertson J, Yong I, Ziebell K, Gannon VPJ (2021). ECTyper: in silico Escherichia coli serotype and species prediction from raw and assembled whole-genome sequence data. Microb Genom.

[68] Inouye M, Dashnow H, Raven LA, Schultz MB, Pope BJ, Tomita T (2014). SRST2: Rapid genomic surveillance for public health and hospital microbiology labs. Genome Med.

[69] Ishijima N, Lee KI, Kuwahara T, Nakayama-Imaohji H, Yoneda S, Iguchi A (2017). Identification of a New Virulent Clade in Enterohemorrhagic *Escherichia coli* O26:H11/H- Sequence Type 29. Sci Rep.

[70] Nakamura K, Tokuda C, Arimitsu H, Etoh Y, Hamasaki M, Deguchi Y (2021). Development of a homogeneous time-resolved FRET (HTRF) assay for the quantification of Shiga toxin 2 produced by *E. coli*. PeerJ..

[71] Okuno M, Arimizu Y, Miyahara S, Wakabayashi Y, Gotoh Y, Yoshino S, et al. Genomic analysis of cryptic *Escherichia* clade I. GenBank https://www.ncbi.nlm.nih.gov/bioproject/727999 (2021).

