# Supplementary material for: Escherichia cryptic clade I is an emerging source of human intestinal pathogens
Source: BMC Biol. 2023 Apr 13;21:81. doi: 10.1186/s12915-023-01584-4 (PMC10100065; doi:10.1186/s12915-023-01584-4)
Supplement: Supplementary file 1 — Additional file 1: Figure S1. Core gene-based phylogenomic tree of the strains in EnteroBase that were suspected to belong to Escherichia cryptic clades and recently defined Escherichia species. Figure S2. Agarose gel electrophoresis analysis of the products obtained by C-I detection PCR. Figure S3. Continents, sources and serotype distributions of strains belonging to C-I, other cryptic clades and recently defined Escherichia species. Figure S4. Summary of the prevalence of virulence genes in C-I, other cryptic clades and recently defined Escherichia species. Figure S5. Distribution of AMR genes in STEC-type, ETEC-type, STEC/ETEC hybrid-type and nonintestinal pathogenic C-I strains. Figure S6. Summary of the prevalence of AMR genes in C-I. Figure S7. Distribution of AMR genes in C-I, other cryptic clades and recently defined Escherichia species. Figure S8. Summary of the prevalence of AMR genes in C-I strains from humans and livestock animals. Figure S9. Distribution of AMR genes in STEC-type, ETEC-type, STEC/ETEC hybrid-type and nonintestinal pathogenic C-I strains. Figure S10. Comparison of genes in the K88-like CF loci with those in the K88 CS locus. Figure S11. Circular map of the plasmid encoding multiple AMR genes and the sepA gene in the C-I strain 10290. [file 12915_2023_1584_MOESM1_ESM.pdf]

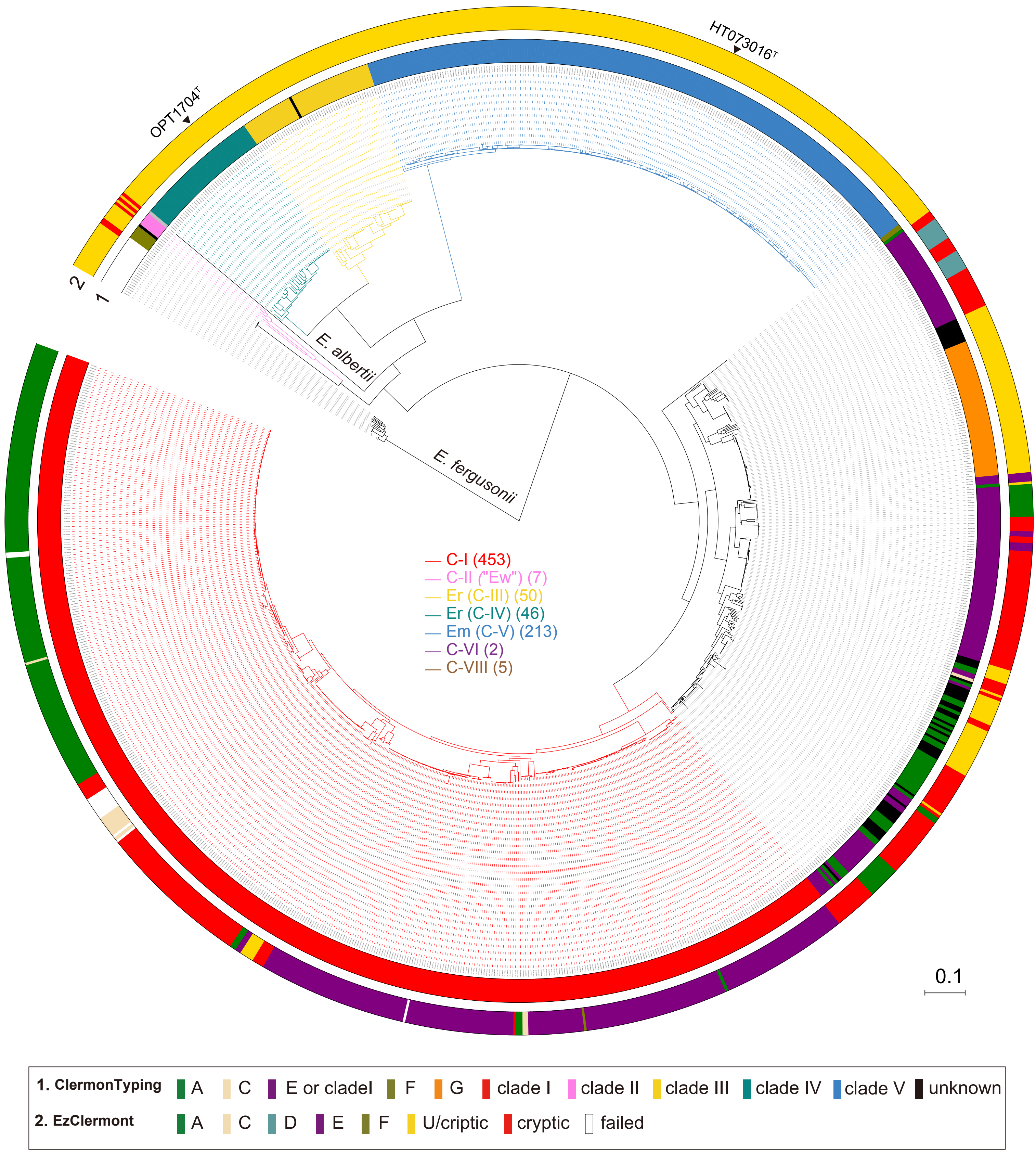

Figure S1. Core gene-based phylogenomic tree of the strains in EnteroBase that were suspected to belong to *Escherichia* cryptic clades and recently defined *Escherichia* species.
A core gene-based ML tree of 1,065 *Escherichia* spp. genomes that were deposited in EnteroBase, do not belong to either *E. fergusonii* or *E. albertii* and are not assigned to any of the *E. coli* phylogroups by both ClermonTyping and EzClermont are shown. Strains classified as cryptic clades and recently defined *Escherichia* species are highlighted by differently coloured lines. *E. coli* phylotype and clade assignments of strains by EzClermont and ClermonTyping were obtained from EnteroBase and are indicated in the outer rings.

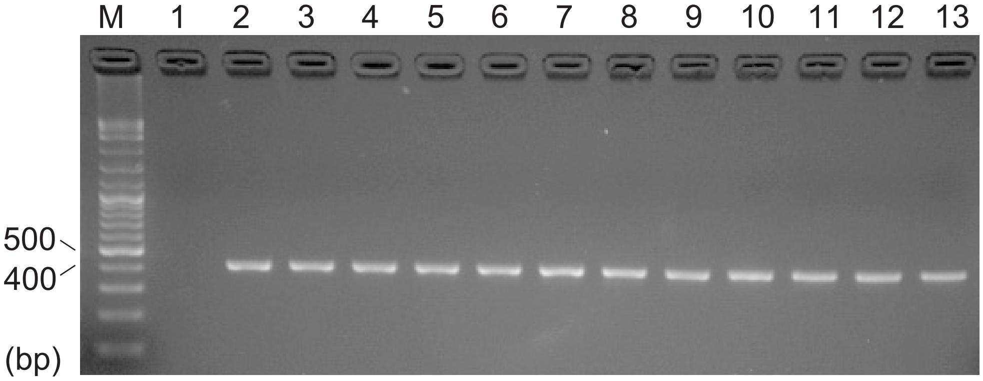

Figure S2. Agarose gel electrophoresis analysis of the products obtained by C-I detection PCR. Twelve C-I strains with a negative control (*E. coli*) were analysed by C-I detection PCR using the primer pair designed in this study. In all the C-I strains, the PCR product of the expected size (400 bp) was obtained as a single band. Lanes M: DNA size marker, 1: E. coli K-12, 2: HH-P024, 3: HH-P041, 4: HH-P049, 5: KS-P062, 6: KS-P079, 7: KS-P095, 8: SI-P041, 9: 10290, 10: EC05-109, 11: PV12-13, 12: PV13-104, 13: PV14-161.

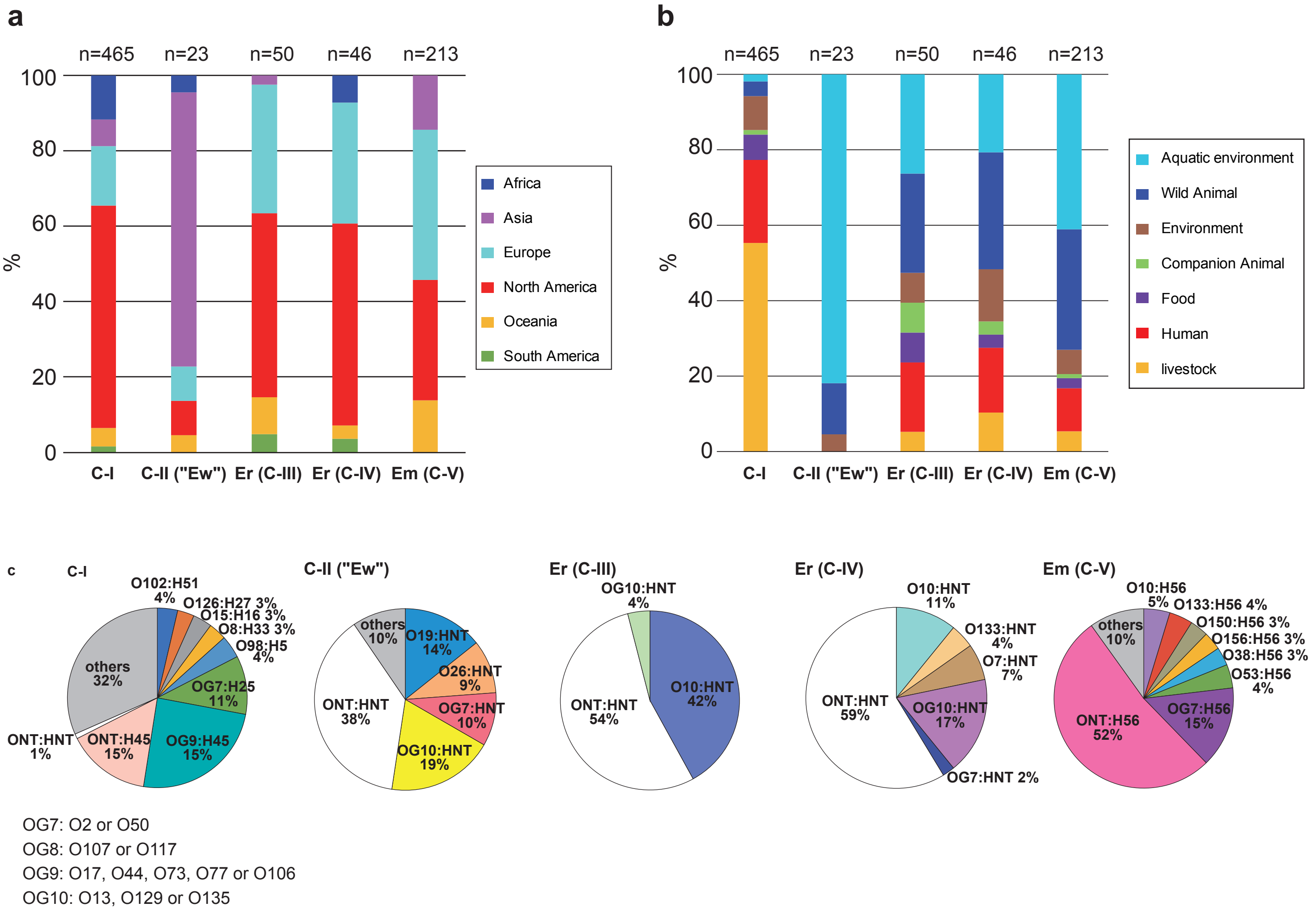

Figure S3. Continents, sources and serotype distributions of strains belonging to C-I, other cryptic clades and recently defined *Escherichia* species.
Summaries of continents (**a**), sources (**b**) and serotype distributions (**c**) of the strains belonging to each phylogenetic group are shown.

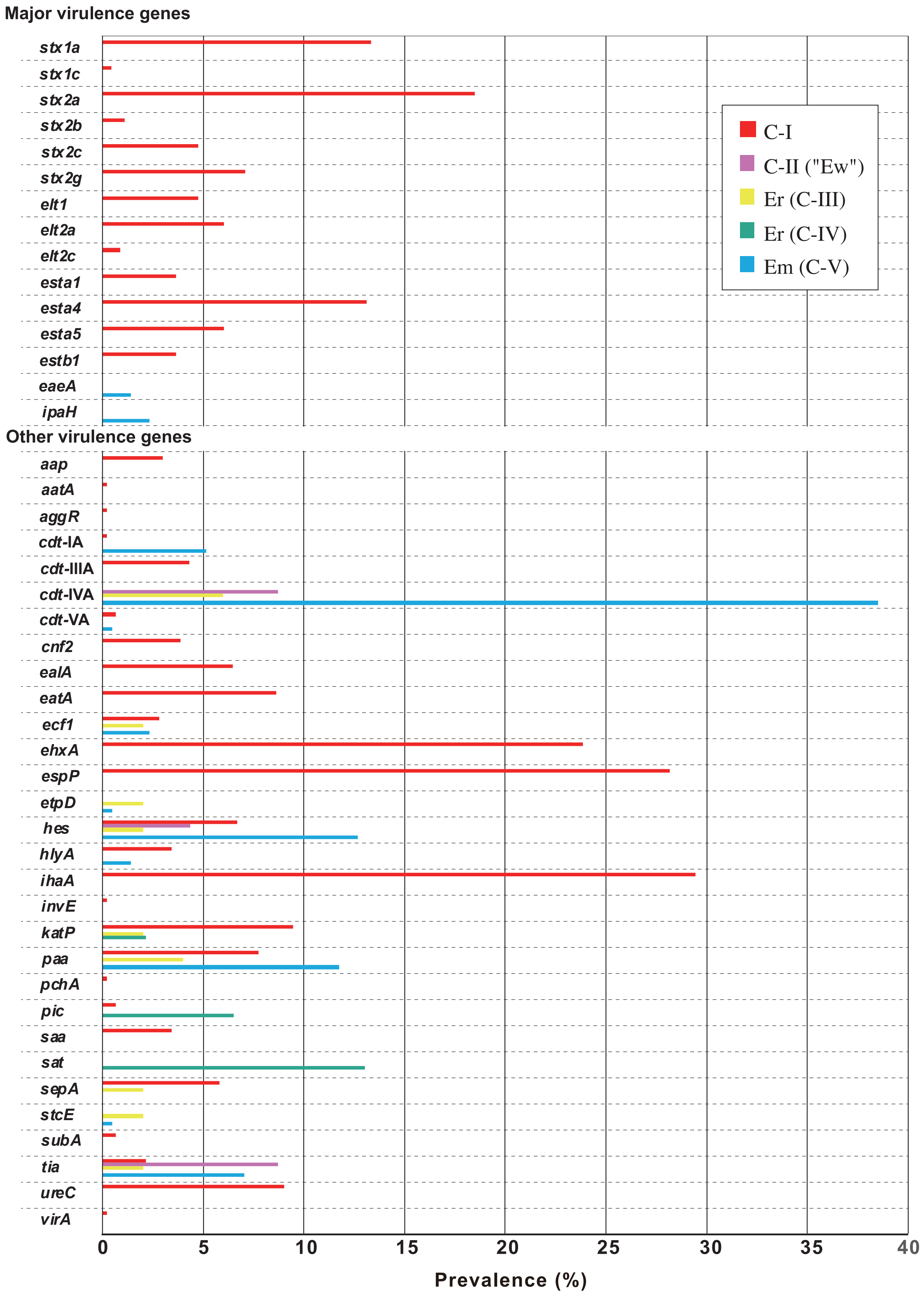

Figure S4. Summary of the prevalence of virulence genes in C-I, other cryptic clades and recently defined *Escherichia* species.

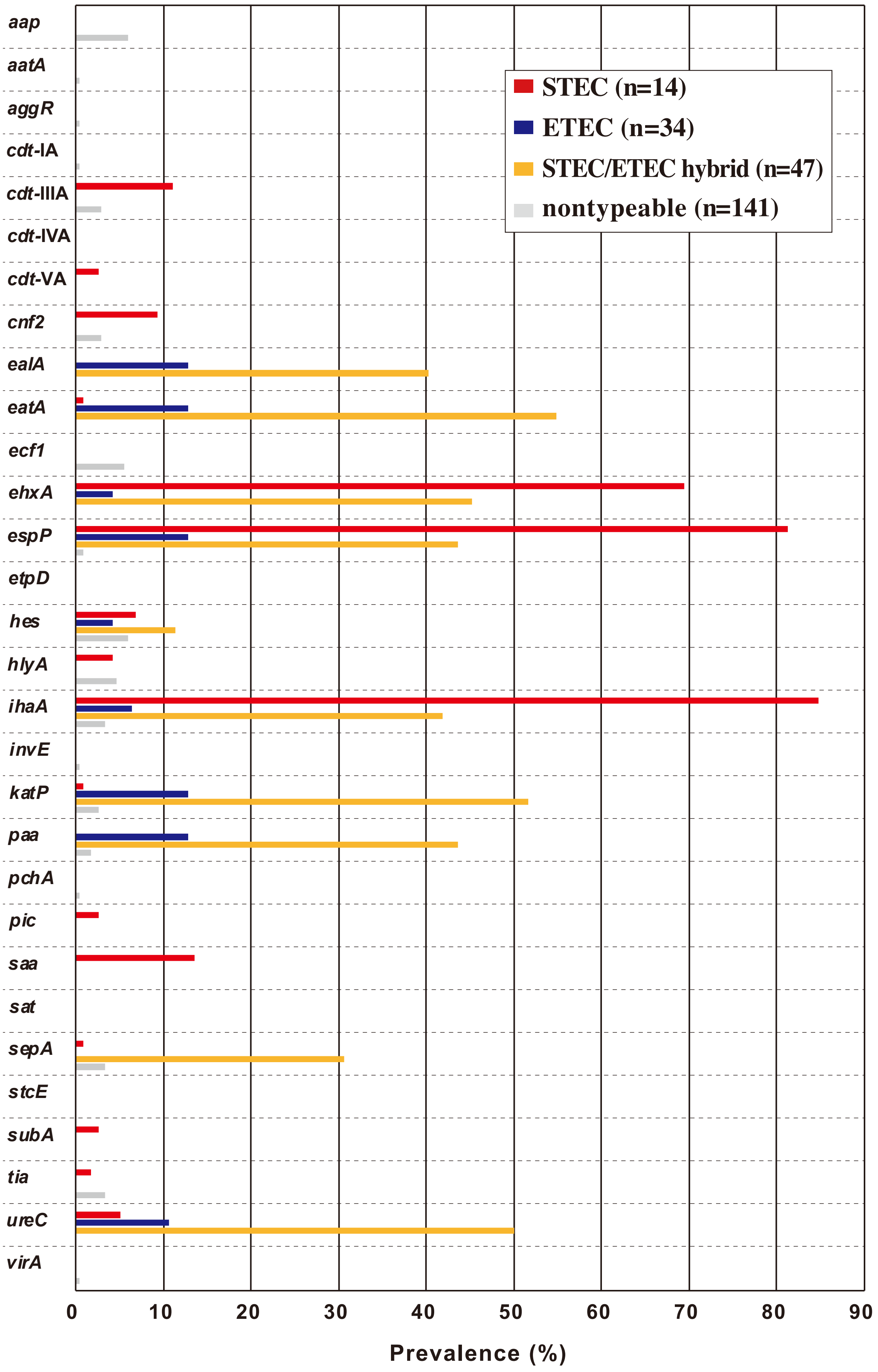

Figure S5. Distribution of AMR genes in STEC-type, ETEC-type, STEC/ETEC hybrid-type and nonintestinal pathogenic C-I strains.

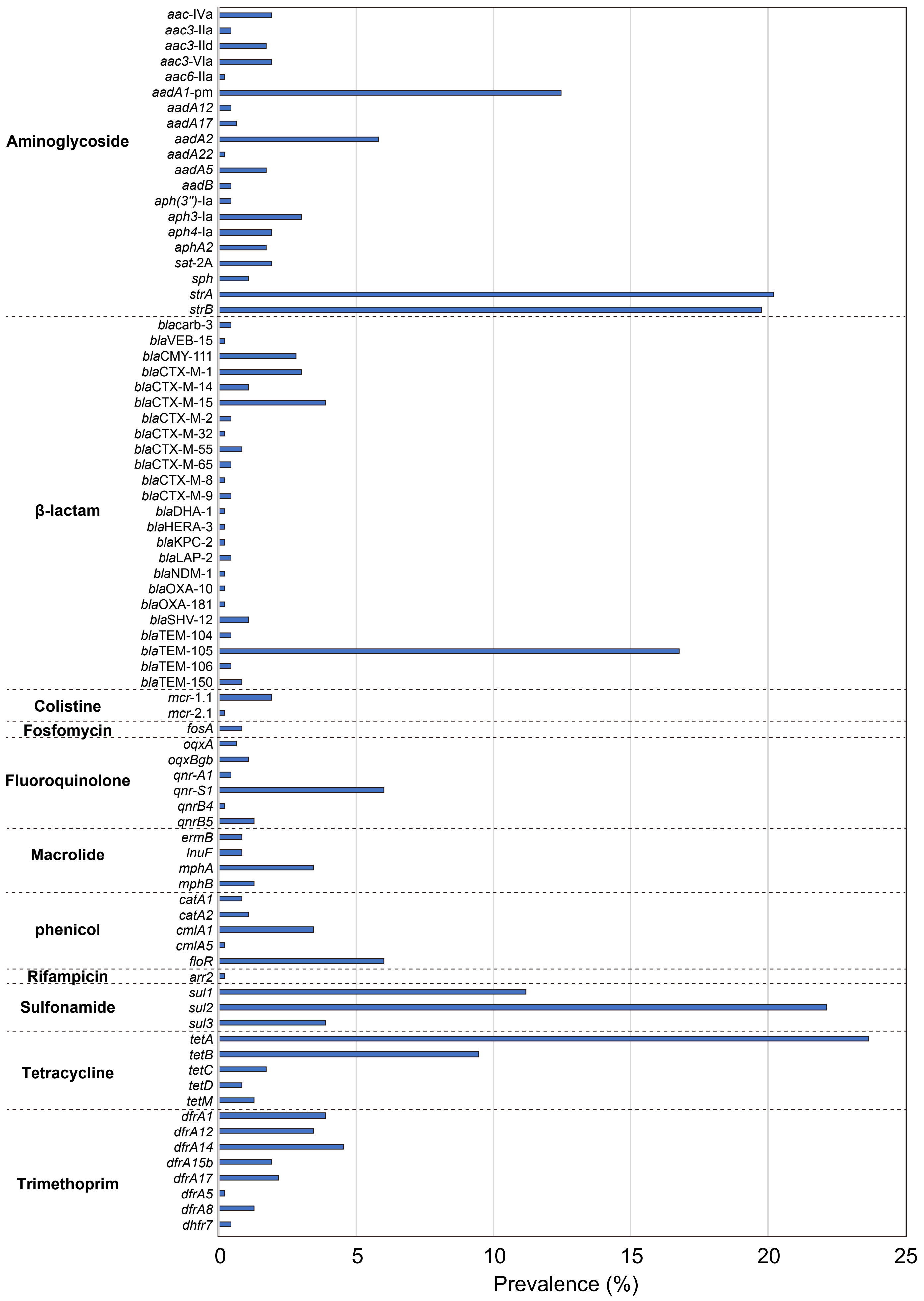

Figure S6. Summary of the prevalence of AMR genes in C-I.

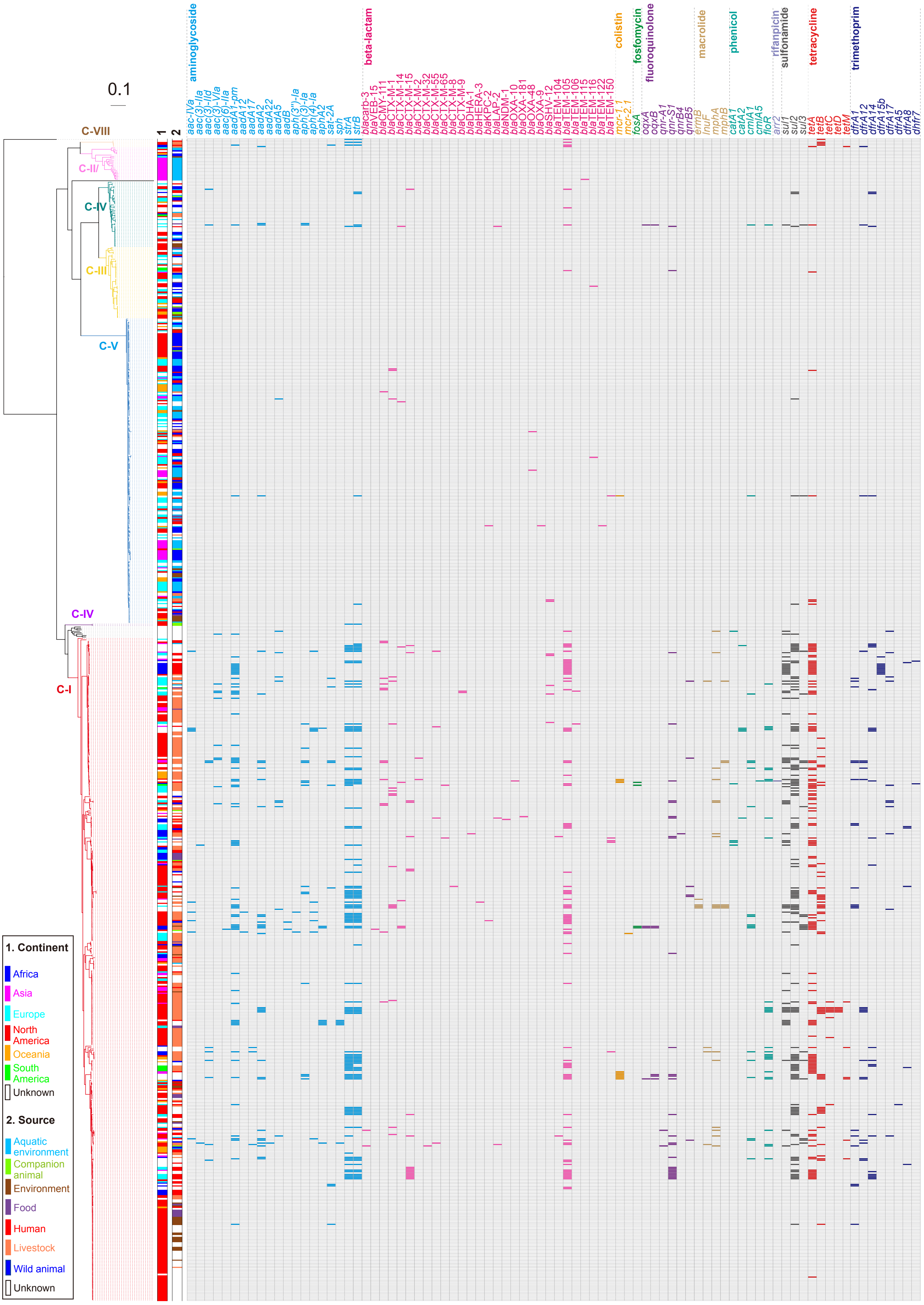

Figure S7. Distribution of AMR genes in C-I, other cryptic clades and recently defined *Escherichia* species.

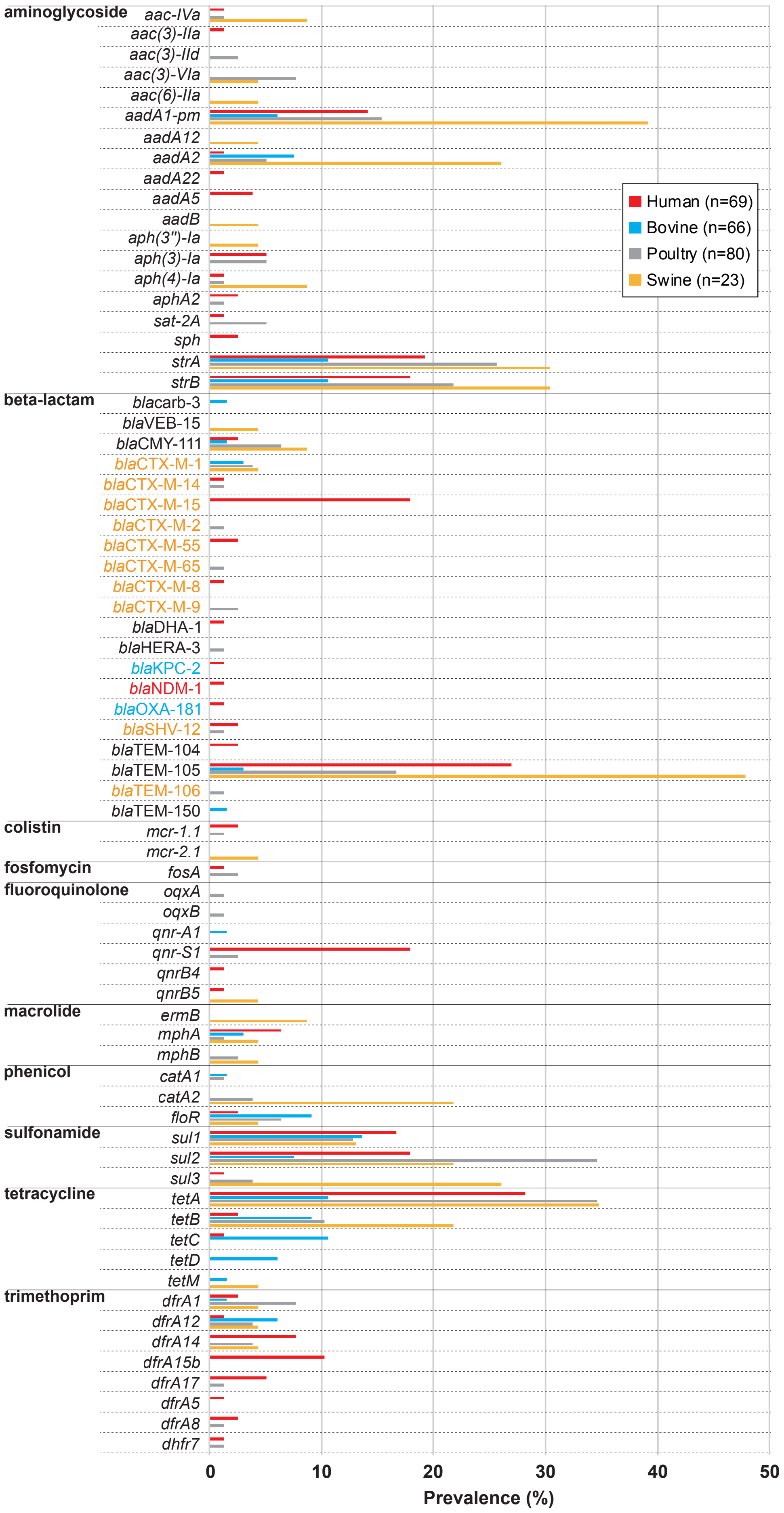

Figure S8. Summary of the prevalence of AMR genes in C-I strains from humans and livestock animals.

Genes for metallo-β-lactamase and other carbapenemases are indicated by red and light blue characters, respectively, and those for ESBLs are indicated by orange characters.

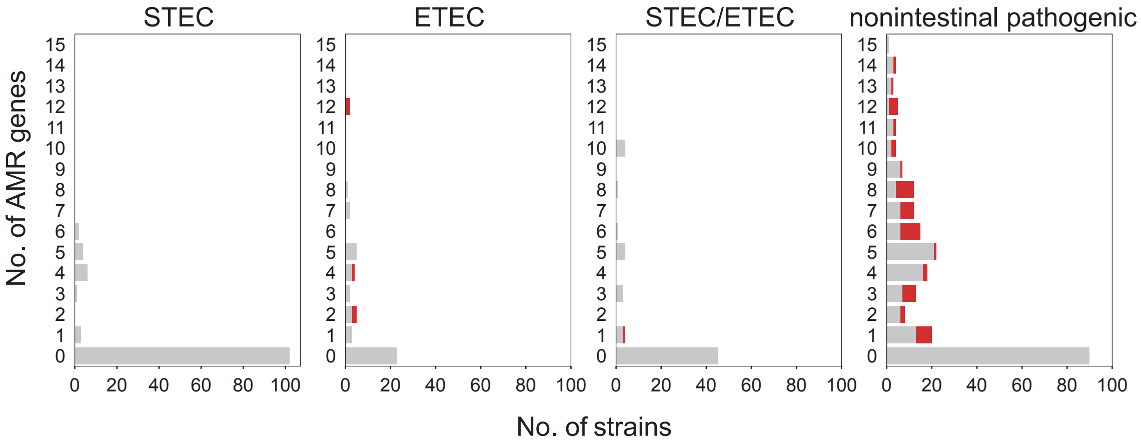

Figure S9. Distribution of AMR genes in STEC-type, ETEC-type, STEC/ETEC hybrid-type and nonintestinal pathogenic C-I strains.
Strains carrying genes for metallo-β-lactamase, other carbapenemases and ESBLs are highlighted in red.

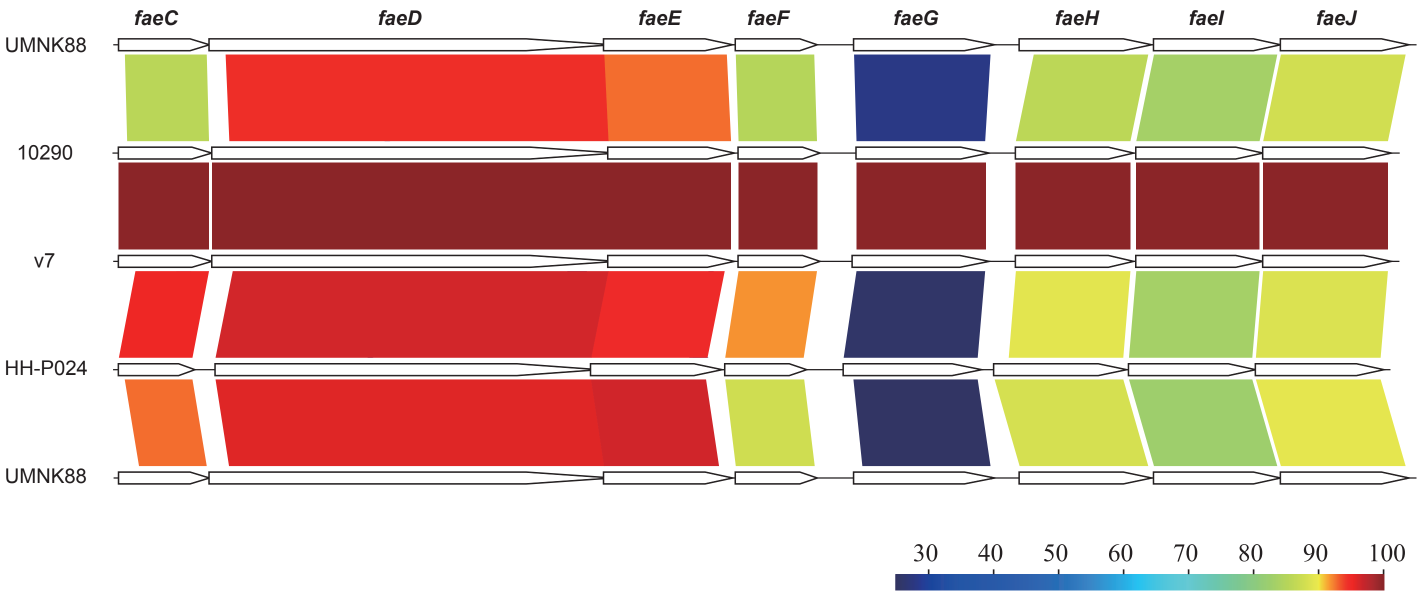

Figure S10. Comparison of genes in the K88-like CF loci with those in the K88 CS locus.
The genetic structures of K88-like CF-encoding loci in C-I strains 10290, v7 and HH-P024 and that of the K88 CS-encoding locus in ETEC strain UMNK88 are drawn to scale. Homologous regions are indicated by shading, and sequence identities are indicated by different colours.

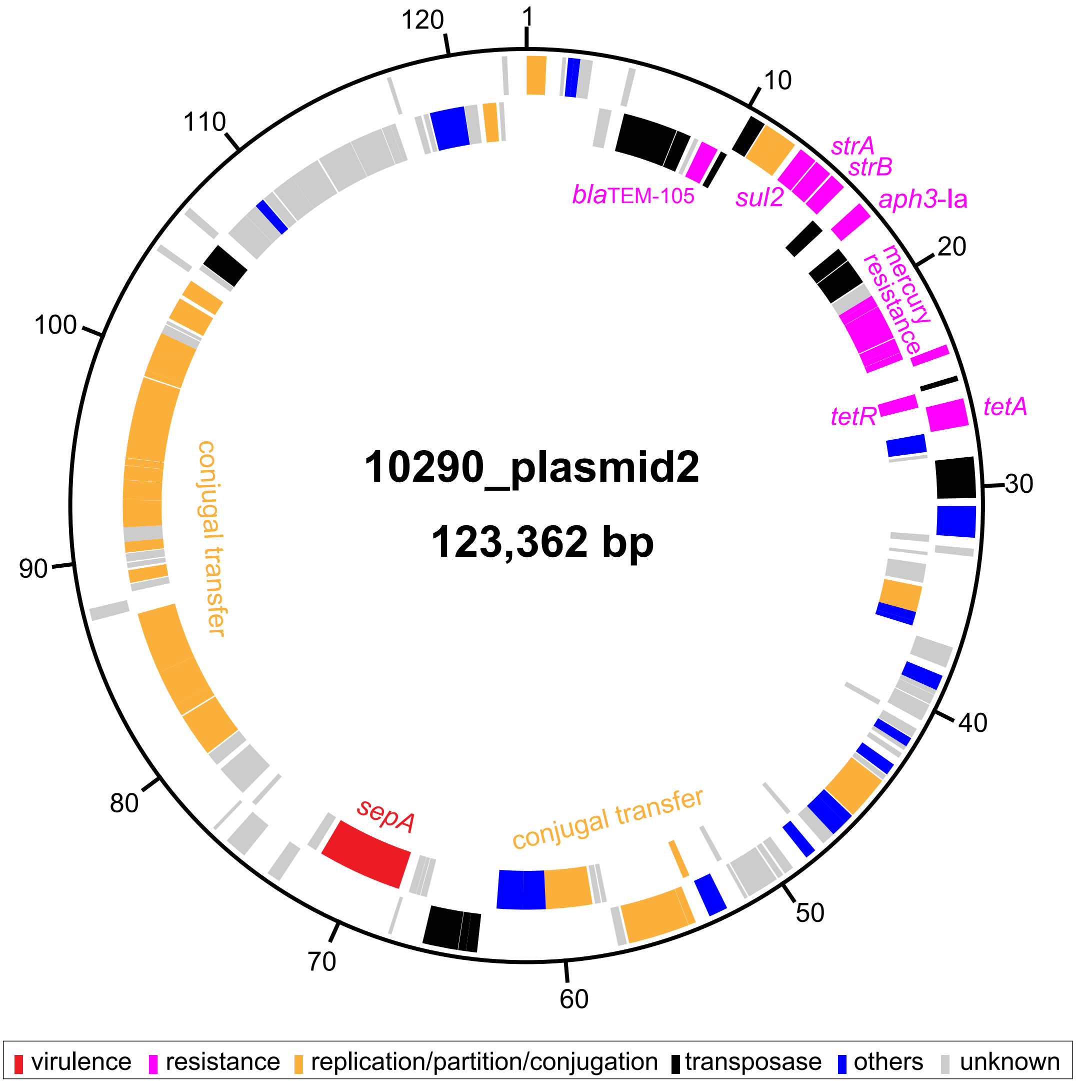

Figure S11. Circular map of the plasmid encoding multiple AMR genes and the sepA gene in the C-I strain 10290.
