# Supplementary material for: Escherichia cryptic clade I is an emerging source of human intestinal pathogens
Source: BMC Biol. 2023 Apr 13;21:81. doi: 10.1186/s12915-023-01584-4 (PMC10100065; doi:10.1186/s12915-023-01584-4)
Supplement: Supplementary file 3 — Additional file 3: Supplementary Text. Colonization factors in C-I strains and structural comparisons of the Stx and LT phages and the virulence plasmids in C-I strains. [file 12915_2023_1584_MOESM3_ESM.pdf]

Escherichia cryptic clade I is an emerging source of human intestinal pathogens

Miki Okuno, Yoko Arimizu, Seina Miyahara, Yuki Wakabayashi, Yasuhiro Gotoh, Shuji Yoshino, Tetsuya Harada, Kazuko Seto, Takeshi Yamamoto, Keiji Nakamura, Tetsuya Hayashi, and Yoshitoshi Ogura

Supplementary Text

Colonization factors in C-I strains

In the ETEC- and STEC/ETEC hybrid-type strains (*est*- and/or *elt*-positive; n=109), known ETEC colonization factors (CFs) were detected in only 17 strains (Additional file 2: Table S1), although various CFs have been identified in human and animal ETEC strains [27, 28]. However, as CFs homologous to the K88 fimbria (also designated the F4 fimbria), which has been shown to be involved in colonization in the small intestines of piglets [28], were previously identified on an *est*-encoding plasmid (p7v) of a C-I strain [23], we performed a search for K88-like fimbria genes and identified two types of K88-like CFs in 41 C-I strains (Additional file 2: Table S1).

In the comparison of virulence plasmids among the completely sequenced C-I strains, the K88 fimbria-like CF of strain HH-P024 showed considerable sequence diversity compared to those of strains 10290/KS-P079/7v, especially in the *faeG* gene encoding the major

subunit/adhesin (Supplementary Fig. 10). The K88 fimbria-like CFs in 10290/KS-P079/7v (named K88-like_10290) and HH-P024 (K88-like_HH-P024) were identified in 35 and 7 C-I strains, respectively (Additional file 2: Table S1).

Among CFs identified in C-I strains, CS12, CS23 and CS28B were specifically detected in human isolates, and K88-like CFs were mostly detected in bovine isolates (Fig. 2c). This may suggest that these CFs are involved in host adaptation in C-I strains.

Structural comparisons of the Stx and LT phages and the virulence plasmids in C-I strains

The Stx1a phages of strains 89-3506 and 2013C-4282 were totally different in genomic structure and similar to the Stx2a phage of STEC O113:H21 strain EH41 [29] and the Stx1a phage of STEC O119:H4 strain 2009C-3133 [31], respectively. As it is widely recognized that Stx phages exhibit substantial genetic variability among STEC strains [32], among the 62 C-I strains positive for *stx1a*, the draft genome sequences covered more than 70% of the Stx1a phage genomes of strains 89-3506 and 2013C-4282 in only 10 strains each (Additional file 2: Table S3). Although the integration sites of the Stx2a phages of two C-I strains (10290 and KS-P079) were different (*wrbA* and *argW*, respectively), their genomic structures were similar to each other. The late regions of these Stx2a phages were also similar to the Stx2a phage of the STEC O157:H7 strain EDL933, which was isolated from a haemorrhagic colitis outbreak in

1983 in Michigan [33]. The Stx2g phage of strain HH-P024 showed the highest similarity to the Stx2g phage of the STEC O36:H14 strain 06-00048 and shared the same integration site (*ssrA*). As like in Stx1a phage, among the 87 C-I strains positive for *stx2a*, the draft genome sequences covered over 70% region of the Stx2a phage genomes of strains 10290 and KS-P079 in only 11 and 8 strains, respectively. Contrary, in 28 of the 33 C-I strains positive for *stx2g*, the draft genome sequences covered over 70% region of the Stx2g phage of strains HH-P024, implying that Stx2g phages are relatively homogeneous in C-I strains.

The LT2a phage in strain KS-P079 showed high similarity to the LT2a phage in the ETEC strain SA53 [30]. The LT2a phage of strain 10290 shared a nearly identical early region with that in strain KS-P079, but a Mu-like phage was integrated into the late region of the phage in strain 10290. In 17 strains among the 33 C-I strains positive for *elt2*, the draft genome sequences covered over 70% region of one of the these completely sequenced LT2 phage genomes (Additional file 2: Table S3). In ETEC, the *elt1* gene is encoded by a plasmid. However, we could not analyse the genomic location of *elt1* in C-I strains because the C-I strains available in our laboratories were all *elt1* negative, and the complete genome sequences of *elt1*-positive C-I strains are not currently available in the public databases.

The entire sequences of the virulence plasmids in strains 10290 and KS-P079 were very similar to that of p7v but showed some differences from that of HH-P024 (Figs. 5b and

5c). In most C-I strains positive for *esta4* and/or *esta5* (57 out of 63), the draft genome sequences covered over 70% region of either the virulence plasmids in strains 10290 or HH-p024, suggesting the origins of virulence plasmids carrying *esta4* and/or *esta5* are shared among C-I strains (Additional file 2: Table S3).
